# Supplementary material for: Squeezing formaldehyde into C60 fullerene
Source: Nat Commun. 2024 Mar 21;15:2515. doi: 10.1038/s41467-024-46886-5 (PMC10957948; doi:10.1038/s41467-024-46886-5)
Supplement: Supplementary file 1 — Supplementary Information [file 41467_2024_46886_MOESM1_ESM.pdf]

Squeezing formaldehyde into C_{60} fullerene.

Vijyesh K. Vyas, George R. Bacanu, Murari Soundararajan, Elizabeth S. Marsden, Tanzeeha Jafari, Anna Shugai, Mark E. Light, Urmas Nagel, Toomas Rõõm,* Malcolm H. Levitt,* Richard J. Whitby.*

Supplementary information.

Contents

1. Characterisation data and pictures of NMR's.

1.1 CH_2O@**2**, $^{13}CH_2O$@**2**, CD_2O@**2**

1.2 CH_2O@**4**, $^{13}CH_2O$@**4**, CD_2O@**4**

1.3 CH_2O@C_{60}, $^{13}CH_2O$@C_{60}, CD_2O@C_{60}

1.4 CH_2O@**6**

1.5 CO_2@**2**

2. 2.1 Kinetics of loss of CH_2O from CH_2O@1 and CH_2O@2.

2.2 Kinetics of loss of CO_2 from CO_2@2.

3. Electrochemistry of CH_2O@C_{60}

4. DFT calculations

4.1 Binding energies and activation energies for entry / exit of CH_2O, H_2O and CO_2 into **1** and **2**.

4.2 Properties of CH_2O@C_{60}.

4.2.1 Minimised structures of CH_2O@C_{60} and C_{60}.

4.2.2 Molecular orbital energies of CH_2O@C_{60} and C_{60}.

4.2.3 Translational modes of CH_2O@C_{60}.

4.2.4 Calculation of excited state energies.

4.2.5 NMR calculations on CH_2O@C_{60} and C_{60}.

5. NMR studies on CH_2O@C_{60}

5.1 ^{1}H spin-lattice relaxation of $^{13}CH_2O$@C_{60}.

5.2 ^{13}C spin-lattice relaxation of CH_2O@C_{60}.

5.3 ^{1}H spin-lattice relaxation of CH_2O@**2** and monomeric CH_2O.

6. THz and IR studies on CH_2O@C_{60}.

7. X-ray structure of CH_2O@C_{60} nickel(II) octaethylporphyrin complex.

8. Photochemistry apparatus.

9. References

List of Supplementary Figures and Tables.

Supplementary Fig. 1. ^{1}H NMR of mixture of CH_2O@**2** and H_2O@**2 + 2.**
Supplementary Fig. 2. ^{13}C NMR of mixture of CH_2O@**2** and H_2O@**2.**
Supplementary Fig. 3. ^{13}C NMR of H_2O@**2.**
Supplementary Fig. 4. ^{1}H NMR of mixture of CH_2O@**3** and H_2O@**3**.
Supplementary Fig. 5. ^{1}H NMR of mixture of CH_2O@**4** and H_2O@**4** in $CDCl_3$.
Supplementary Fig. 6. ^{1}H NMR of mixture of CH_2O@**4** and H_2O@**4** in *o*-$C_6D_4Cl_2$-*d_4***.**
Supplementary Fig, 7. ^{13}C NMR of CH_2O@**4** and H_2O@**4.**
Supplementary Fig. 8. ^{1}H NMR of mixture of CH_2O@**5** and H_2O@**5**.
Supplementary Fig. 9. ^{1}H NMR of $CH_2O@C_{60}$.
Supplementary Fig. 10. ^{13}C NMR of $CH_2O@C_{60}$
Supplementary Fig. 11. FT-IR comparison for C_{60} and $CH_2O@C_{60}$
Supplementary Fig. 12. UV-vis spectra of $CH_2O@C_{60}$ and C_{60}.
Supplementary Fig. 13. ^{1}H NMR of CH_2O@**6** and H_2O@**6**.
Supplementary Fig. 14. ^{13}C NMR of CH_2O@**6** and H_2O@**6.**
Supplementary Fig. 15. ^{1}H NMR of CO_2@**2** + H_2O@**2**.
Supplementary Fig. 16. ^{13}C NMR of CO_2@**2**.
Supplementary Fig. 17. 1st order plots for loss of CH_2O from CH_2O@**1** at 25 °C and 40 °C, loss of CH_2O from CH_2O@**2** at 55 °C and loss of CO_2 from CO_2@**2** at 55 °C.
Supplementary Fig. 18. Cyclic Voltammetry of C_{60} and $CH_2O@C_{60}$.
Supplementary Fig. 19. Minimum energy conformers of $CH_2O@C_{60}$**.**
Supplementary Fig. 20. HOMO and LUMO of $CH_2O@C_{60}$ 66 iso.
Supplementary Fig. 21. ^{1}H T_1 relaxation curves (Inversion Recovery) of $^{13}CH_2O@C_{60}$ ^{1}H satellites
Supplementary Fig. 22. ^{13}C T_1 relaxation curve (Inversion Recovery) of CH_2O@<u>C</u>$_{60}$
Supplementary Fig. 23. ^{1}H T_1 relaxation curve (Inversion Recovery) of CH_2O@**2**
Supplementary Fig. 24. ^{1}H T_1 relaxation curve (Inversion Recovery) of monomeric CH_2O
Supplementary Fig. 25. Comparison of transmission spectra of $CH_2O@C_{60}$ and 4He@C60.
Supplementary Fig. 26. Absorption spectrum of $CH_2O@C_{60}$ at 5K and ortho-para conversion of $CH_2O@C_{60}$ at 5K.
Supplementary Fig. 27. DFT minimised position of the CH_2O in the X-ray structure of $CH_2O@C_{60}$ nickel(II) octaethylporphyrin complex.
Supplementary Fig. 28. Single crystal x-ray structure of the nickel(II) octaethylporphyrin/benzene solvate of $CH_2O@C_{60}$
Supplementary Fig. 29. Custom built photochemistry apparatus.

Supplementary Table 1. Loss of CH_2O from CH_2O@**1**
Supplementary Table 2. Loss of CH_2O from CH_2O@2 at 55 °C
Supplementary Table 3. Loss of CO_2 from CO_2@2 at 55 °C
Supplementary Table 4. Reduction potential of C_{60} and $CH_2O@C_{60}$
Supplementary Table 5. DFT energies for complexation in Hartree.
Supplementary Table 6. Calculated free energies of binding, entry and exit of the endohedral species.
Supplementary Table 7. Diameters of $CH_2O@C_{60}$ and C_{60}.
Supplementary Table 8. Calculated diameters of $CH_2O@C_{60}$ and C_{60} cages.
Supplementary Table 9. Calculated Molecular Orbital Energies of $CH_2O@C_{60}$ and C_{60}.
Supplementary Table 10. Calculated rotational and translational modes of CH_2O in $CH_2O@C_{60}$.
Supplementary Table 11. Singlet excited state energies for $CH_2O@C_{60}$ and C_{60}.

Supplementary Table 12. Calculated ^{13}C NMR shifts for C_{60} and $CH_2O@C_{60}$
Supplementary Table 13. Summary of ^{13}C calculated Chemical shift changes on CH_2O incorporation.

1. Characterisation data.

^{1}H and ^{13}C NMR spectra for all compounds were recorded on Bruker AVIIIHD400 or AVIIIHD500 FT-NMR spectrometers, in the indicated solvent at 298 K. ^{1}H chemical shifts are reported as values in ppm referenced to residual solvent ($CHCl_3$, δ =7.26 ppm, $CHDCl_2$,δ = 5.32 ppm, *o*-$C_6D_3HCl_2$, δ = 6.93 and 7.19 ppm, $C_6D_5CD_2H$, δ = 2.09 ppm) and the following abbreviations are used to assign multiplicity and may be compounded: s = singlet, d = doublet, t = triplet, and m = multiplet. Coupling constants, *J*, are measured in Hertz (Hz). ^{13}C NMR spectra were ^{1}H decoupled except where stated and are referenced to solvents, *o*-$C_6D_4Cl_2$ at δ = 127.19 ppm (centre of the 1:1:1 triplet)[2], $CDCl_3$ at δ = 77.24 ppm (centre of the triplet) and CD_2Cl_2 = 53.84 ppm (central peak of pentet), toluene-d_8, δ = 137.86 ppm (downfield singlet); the solvent chemical shift is referenced to TMS (δ = 0.00 ppm). ^{2}H NMR were taken using deuterated TMS as an external reference but were referenced to natural abundance deuterated solvent $CDCl_3$, δ =7.26 ppm, $C_6H_5CH_2D$, δ = 2.09 ppm.

Negative ion electrospray mass spectra were recorded using a MaXis time of flight (TOF) mass spectrometer (Bruker Daltonik GmbH, Bremen, Germany). IR spectra of $CH_2O@C_{60}$ and C_{60} were obtained by using a Nicolet 380 FTIR instrument with Smart orbit ATR accessory. UV measurements were recorded on an Ocean Optics DH-2000-BAL spectrometer with a 1 cm path length cell.

1.1 CH_2O@2

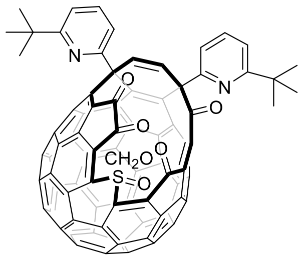

^{1}H NMR (400 MHz, CD_2Cl_2) δ 7.649 (*t*, J = 7.8 Hz, 0.30H, pyridyl of H_2O@**2** and **2**), 7.643 (*t*, J = 7.8 Hz, 0.70H, pyridyl of CH_2O@**2**), 7.635 (*t*, J = 7.9 Hz, 0.30H, pyridyl of H_2O@**2** and **2**), 7.628 (*t*, J = 7.9 Hz, 0.70H, pyridyl of CH_2O@**2**), 7.283-7.127 (m, 5H, pyridyl and alkenyl protons, region mainly obscured by the toluene), 6.715 (d, J = 10.1 Hz, 0.70H, alkenyl in CH_2O@**2**), 6.695 (d, J = 10.1 Hz, 0.30H, alkenyl in H_2O@**2** and **2**), 1.191 (*s*, 2.7H, *t*-butyl for H_2O@**2** and **2**), 1.187 (*s*, 6.3H, *t*-butyl for CH_2O@**2**), 1.165 (*s*, 2.7H, *t*-butyl for H_2O@**2** and **2**), 1.160 (*s*, 6.3H, *t*-butyl for CH_2O@**2**), -1.007 (s, 1.4H, endohedral C**H**$_2$O@2), -10.308 (br s, 0.5H, endohedral **H**$_2$O@2).

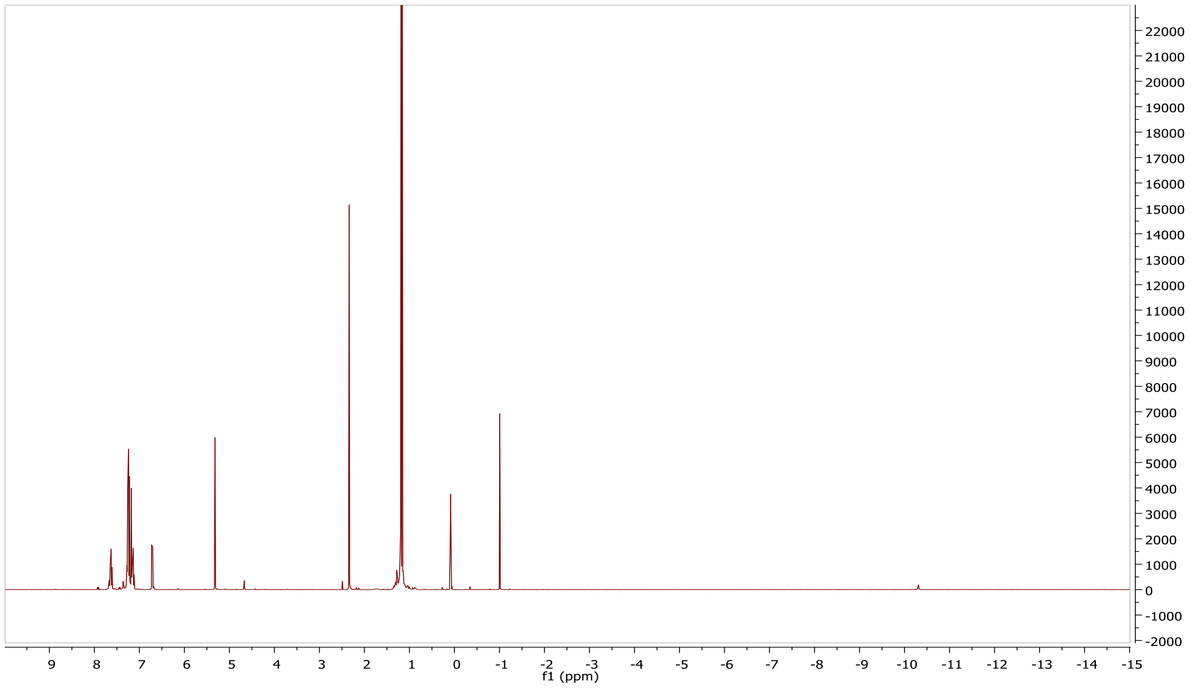

Supplementary Fig. 1. ^{1}H NMR of mixture of CH_2O@**2** and H_2O@**2** + **2.**

^{13}C NMR of CH_2O@2 (101 MHz, CD_2Cl_2) δc 191.86 (C), 186.23 (C), 185.77 (CH2), 184.46 (C), 181.91 (C), 169.17 (C), 168.88 (C), 163.57 (C), 162.45 (C), 156.97 (C), 156.21 (C), 151.64 (C), 151.5 (C), 151.27 (C), 151.03 (C), 150.87 (C), 150.82 (C), 150.8 (C), 150.72 (C), 150.6 (C), 150.53 (C), 150.35 (C), 149.78 (C), 149.68 (C), 148.47 (C), 148.16 (C), 147.86 (C), 147.46

(C), 145.81 (C), 145.64 (C), 145.35 (C), 144.59 (C), 144.47 (C), 144.35 (C), 144.14 (C), 143.94 (C), 143.83 (C), 143.67 (C), 143.64 (C), 143.44 (C), 142.38 (C), 141.82 (C), 141.05 (C), 140.66 (C), 139.95 (C), 139.61 (C), 139.07 (C), 138.66 (C), 138.31 (C), 138.22 (C), 138.16 (C), 138.07 (CH), 137.68 (CH), 137.6 (C), 137.31 (C), 137.07 (C), 137 (C), 136.95 (C), 136.03 (C), 135.59 (C), 133.92 (C), 133.23 (C), 132.76 (C), 131.83 (CH), 130.7 (C), 126.89 (C), 126.42 (C), 126.26 (CH), 120.52 (CH), 120.08 (CH), 118.1 (CH), 117.85 (CH), 60.14 (C), 55.3 (C), 37.98 (C), 37.93 (C), 29.97 (6xCH3). One quaternary peak not identified. The peaks for CH_2O@**2** were identified by comparing with the **^{13}C** spectra of H_2O@**2** (below). There is a toluene impurity which could not be removed without also losing the CH_2O.

^{13}C NMR of H_2O@2 (101 MHz, CD_2Cl_2) δ_C 191.71 (C), 186.21 (C), 184.23 (C), 181.81 (C), 169.16 (C), 168.88 (C), 163.44 (C), 162.35 (C), 156.65 (C), 156.14 (C), 151.48 (C), 151.14 (2 x C), 150.64 (C), 150.62 (C), 150.51 (C), 150.31 (C), 150.16 (C), 150.1 (C), 150.02 (C), 149.75 (C), 149.38 (C), 149.12 (C), 148.2 (C), 148.01 (C), 147.73 (C), 147.52 (C), 145.78 (C), 145.55 (C), 145.18 (C), 144.5 (C), 144.21 (2 x C), 144.16 (C), 143.79 (C), 143.73 (C), 143.61 (C), 143.52 (C), 143.25 (C), 142.37 (C), 141.71 (C), 141.05 (C), 140.3 (C), 139.64 (C), 139.21 (C), 138.83 (C), 138.3 (C), 138.11 (2x C), 138.07 (CH), 137.79 (C), 137.69 (CH), 137.42 (C), 137 (C), 136.82 (C), 136.74 (C), 136.48 (C), 135.05 (C), 134.62 (C), 133.5 (C), 133.02 (C), 132.39 (C), 131.76 (CH), 130.64 (C) , 126.45 (C), 126.21 (CH), 120.48 (CH), 120.05 (CH), 118.12 (CH), 117.85 (CH), 60.03 (C), 55.14 (C), 37.96 (C), 37.9 (C), 29.96 (6 x CH3). 4 quaternary peaks not observed. Many of the peaks are broadened by equilibrium with empty sulfoxide occurring on the NMR timescale. Approximately 10% empty **2** is present but peaks not reported.

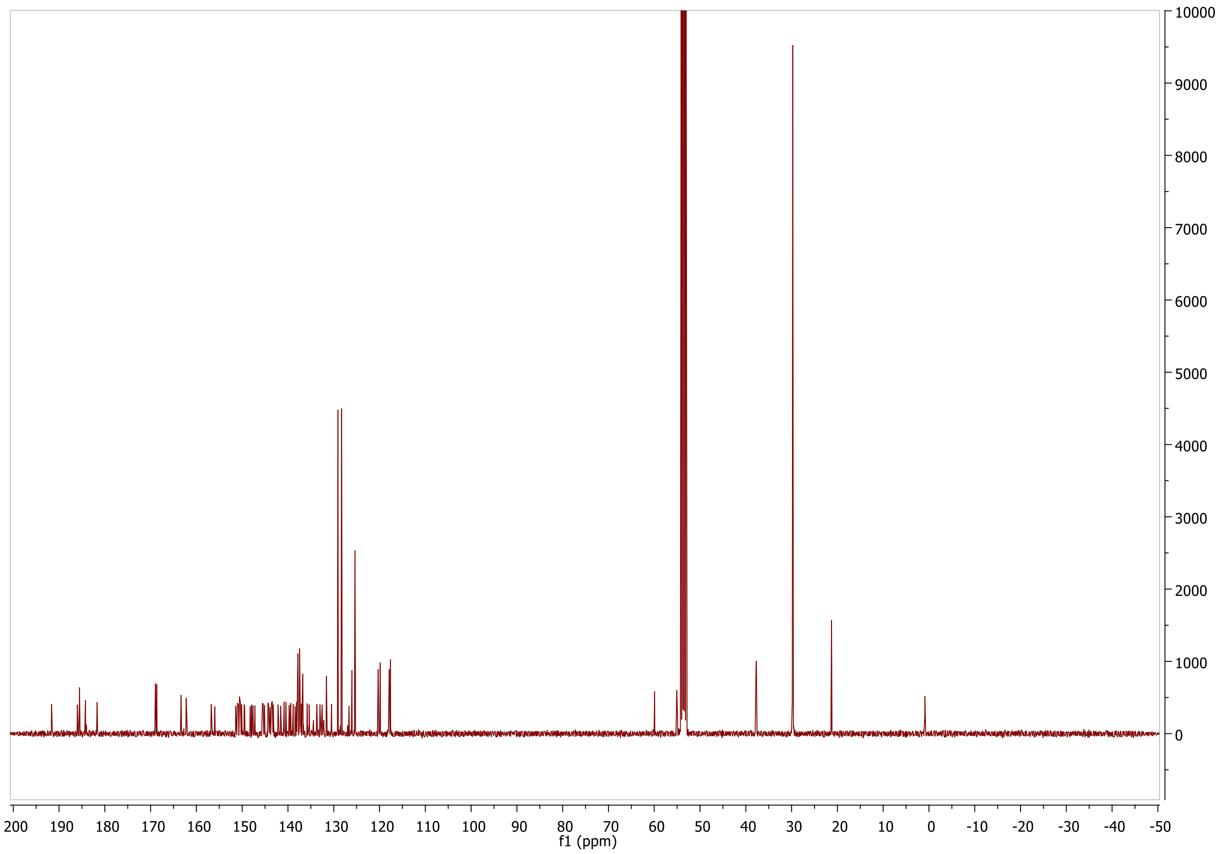

Supplementary Fig. 2. ^{13}**C NMR** of mixture of CH_2O@**2** and H_2O@**2.**

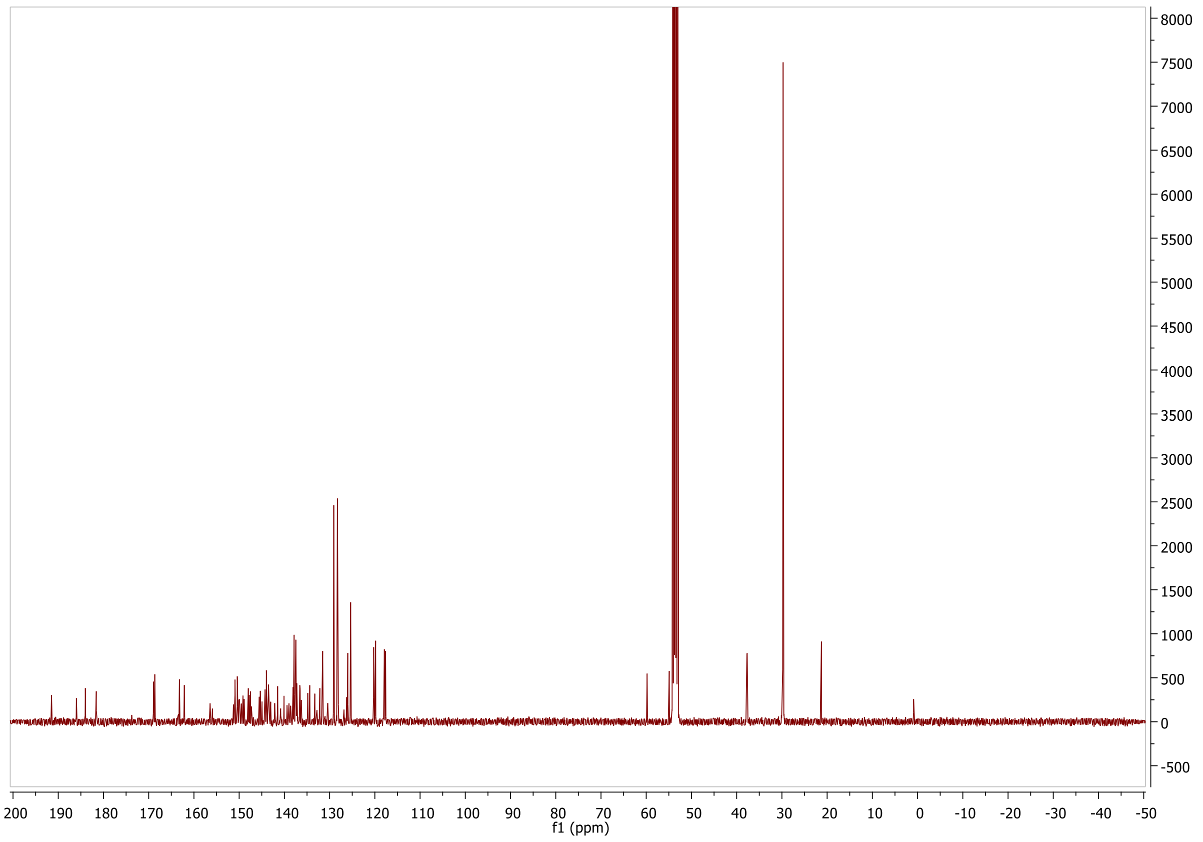

Supplementary Fig. 3. ^{13}**C NMR** of H_2O@**2.**

HRMS (ESI+) (*m/z*): Calculated for $[C_{83}H_{29}N_2O_6S]^+$ 1181.1741, found 1181.1731 (CH_2O filled). Calculated for $[C_{82}H_{27}N_2O_5S]^+$ 1151.1635, found 1151.1628 (empty).

$^{13}CH_2O$@2

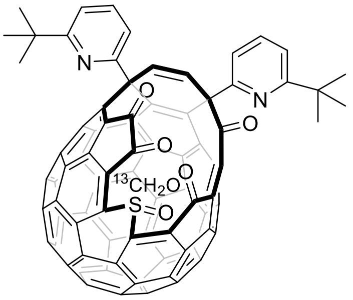

The mixture of $^{13}CH_2O$@**2** and H_2O@**2** and **2** with CH_2O filling of 24%.

^{1}H NMR (400 MHz, $CDCl_3$) δ 7.619 (t, J = 7.8 Hz, 0.76H, pyridyl of H_2O@**2** and **2**), 7.613 (t, J = 7.8 Hz, 0.24H, pyridyl of CH_2O@**2**), 7.602 (t, J = 7.9 Hz, 0.76H, pyridyl of H_2O@**2** and **2**), 7.595 (t, J = 7.9 Hz, 0.24H, pyridyl of CH_2O@**2**), 7.282-7.130 (m, 5H, pyridyl and alkenyl protons, region mainly obscured by the toluene), 6.751 (d, J = 10.3 Hz, 0.24H, alkenyl in CH_2O@**2**), 6.766 (d, J = 10.3 Hz, 0.76H, alkenyl in H_2O@**2** and **2**), 1.179 (*s*, 6.8H, *t*-butyl for H_2O@**2** and **2**), 1.175 (*s*, 2.2H, *t*-butyl for CH_2O@**2**), 1.139 (*s*, 6.8H, *t*-butyl for H_2O@**2** and **2**), 1.135 (*s*, 2.2H, *t*-butyl for CH_2O@**2**), -0.903 (d, J = 174.2 Hz, 0.48H, endohedral C**H**$_2$O@2), -10.280 (br s, 1.3H, endohedral **H**$_2$O@2).

^{13}C NMR (101 MHz, $CDCl_3$, proton decoupled) δc 185.70 ppm (endohedral $^{13}CH_2O$).

HRMS (ESI+) (*m/z*): Calculated for $[^{13}CC_{82}H_{29}N_2O_6S]^+$ 1182.1774, found 1182.1791 ($^{13}CH_2O$@**2**). Calculated for $[C_{82}H_{27}N_2O_5S]^+$ 1151.1635, found 1151.1656 (empty **2**).

CD_2O@2

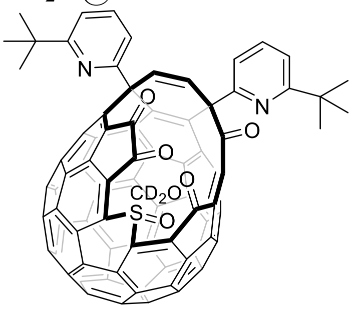

The mixture of CD_2O@**2** and H_2O@**2** + **2** with a ratio of 25:75

^{2}H NMR (77 MHz, $CHCl_3$) δ -0.848 ppm.

HRMS (ESI+) (*m/z*): Calculated for $[C_{83}H_{27}D_2N_2O_6S]^+$ 1183.1866, found 1183.1791 (CD_2O@**2**). Calculated for $[C_{82}H_{27}N_2O_5S]^+$ 1151.1635, found 1151.1646 (empty **2**). Calculated for $[C_{82}H_{29}N_2O_6S]^+$ 1169.1741, found 1169.1734 (H_2O@**2**).

1.2 CH_2O@4

Intermediate CH_2O@**3** (31 : 69 mixture with H_2O@**3**).

^{1}H NMR (400 MHz, $CDCl_3$) δ 7.639 (t, J = 7.9 Hz, 0.31H, pyridyl of H_2O@**3**), 7.631 (t, J = 7.9 Hz, 0.69H, pyridyl of H_2O@**3)**, 7.563 (t, J = 7.8 Hz, 0.69H, pyridyl of H_2O@**3**), 7.557 (t, J = 7.8 Hz, 0.31H, pyridyl of CH_2O@**3**), 7.479 (dd, J = 7.8, 0.8 Hz, 0.31H, pyridyl of CH_2O@**3**), 7.468 (dd, J = 7.8, 0.8 Hz, 0.69H, pyridyl of H_2O@**3**), 7.285 – 7.155 (m, 3H, pyridyl protons, region mainly obscured by the toluene), 7.137 (d, J = 10.1 Hz, 0.31H, alkenyl in CH_2O@**3**), 7.102 (d, J = 10.1 Hz, 0.69H, alkenyl in H_2O@**3**), 7.032 (d, J = 10.1 Hz, 0.31H, alkenyl in CH_2O@**3)** 6.997 (d, J = 10.1 Hz, 0.69H, alkenyl in H_2O@**3**), 1.234 (*s*, 2.8H, *t*-butyl for CH_2O@**3**), 1.227 (*s*, 6.2H, *t*-butyl for H_2O@**3**), 1.168 (*s*, 6.2H, *t*-butyl for H_2O@**3**), 1.163 (*s*, 2.8H, *t*-butyl for CH_2O@**3**), -0.009 (s, 0.62H, endohedral C**H**$_2$O@**3**), -9.852 (s, 1.30H, endohedral **H**$_2$O@**3**).

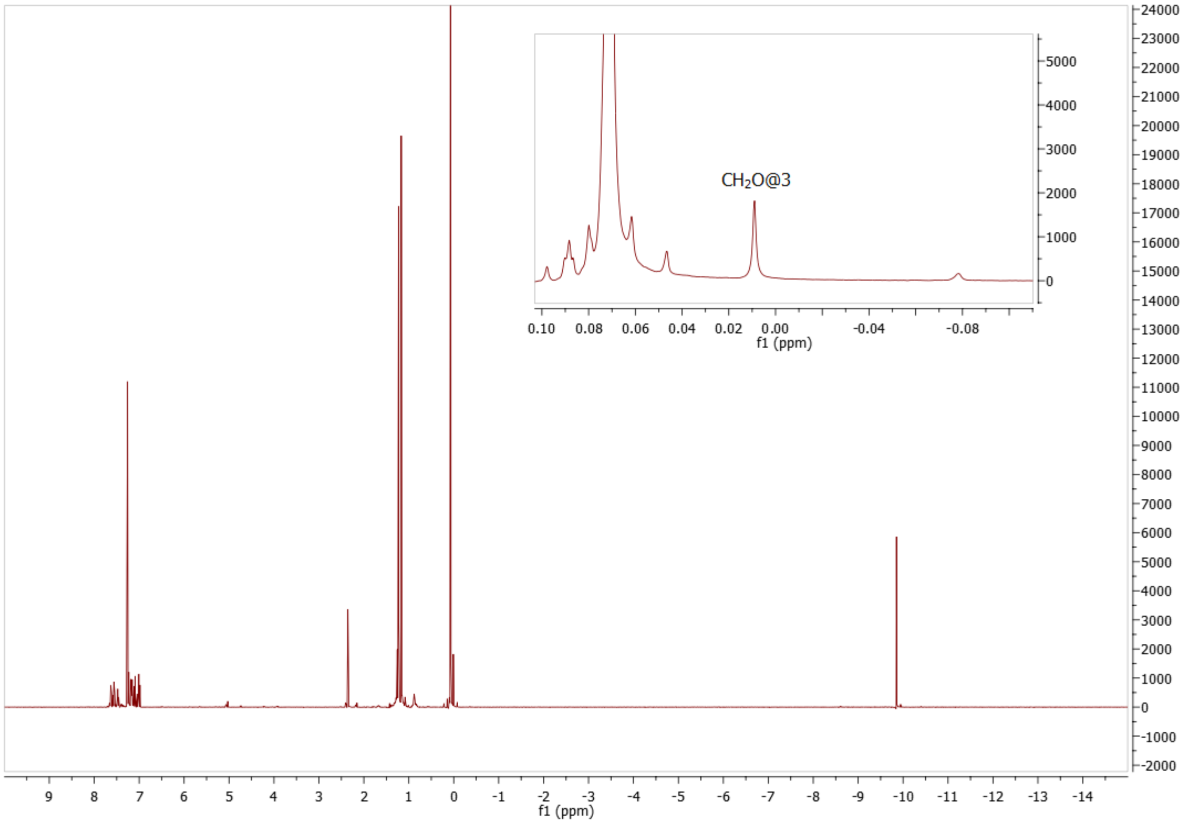

Supplementary Fig. 4. **^{1}H NMR** of mixture of CH_2O@**3** and H_2O@**3**.

CH_2O@**4** (mixture with H_2O@**4** with a ratio of 35:65. (<5% empty **4** also present but could not be quantified)).

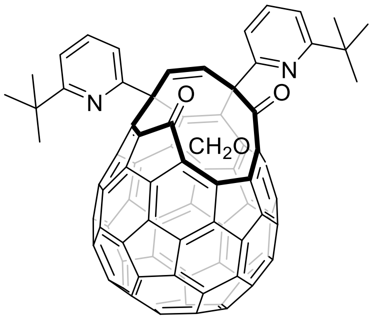

^{1}H NMR (400 MHz, $CDCl_3$) δ 7.697 (t, J = 7.8 Hz, 0.35H, pyridyl for CH_2O@**4**), 7.685 (t, J = 7.8 Hz, 0.65H, pyridyl for H_2O@**4**), 7.657 (t, J = 7.8 Hz, 1H, pyridyl for CH_2O@**4** + H_2O@**4**), 7.531 (dd, J = 7.8 Hz, 0.8 Hz, 0.35H, pyridyl for CH_2O@**4**), 7.512 (dd, J = 7.8 Hz, 0.8 Hz, 0.65H, pyridyl for H_2O@**4**), 7.387 (dd, J = 7.8 Hz, 0.8 Hz, 0.35H, pyridyl for CH_2O@**4**), 7.385 (dd, J = 7.8 Hz, 0.8 Hz, 0.65H, pyridyl for H_2O@**4**), 7.242 (d, J = 10.0 Hz, 0.35H, alkenyl for CH_2O@**4**), 7.231 (dd, J = 7.8 Hz, 0.8 Hz, 2H, pyridyl for CH_2O@**4** + H_2O@**4**), 7.224 (d, J = 10.0 Hz, 0.65H, alkenyl for H_2O@**4**), 7.222 (d, J = 10.0 Hz, 0.35H, alkenyl for CH_2O@**4**),

7.171 (d, J = 10.0 Hz, 0.65H, alkenyl for H_2O@**4**), 1.263 (s, 5.8H, *t*-butyl of H_2O@**4**), 1.260 (s, 3.2H, *t*-butyl of CH_2O@**4**), 1.187 (s, 3.2H, *t*-butyl of CH_2O@**4**), 1.176 (s, 5.8H, *t*-butyl of H_2O@**4** and **4**), 0.803 (s, 0.70H, endohedral C**H**$_2$O@**4**), -8.797 (s, 1.14H, endohedral **H**$_2$O@**4**).

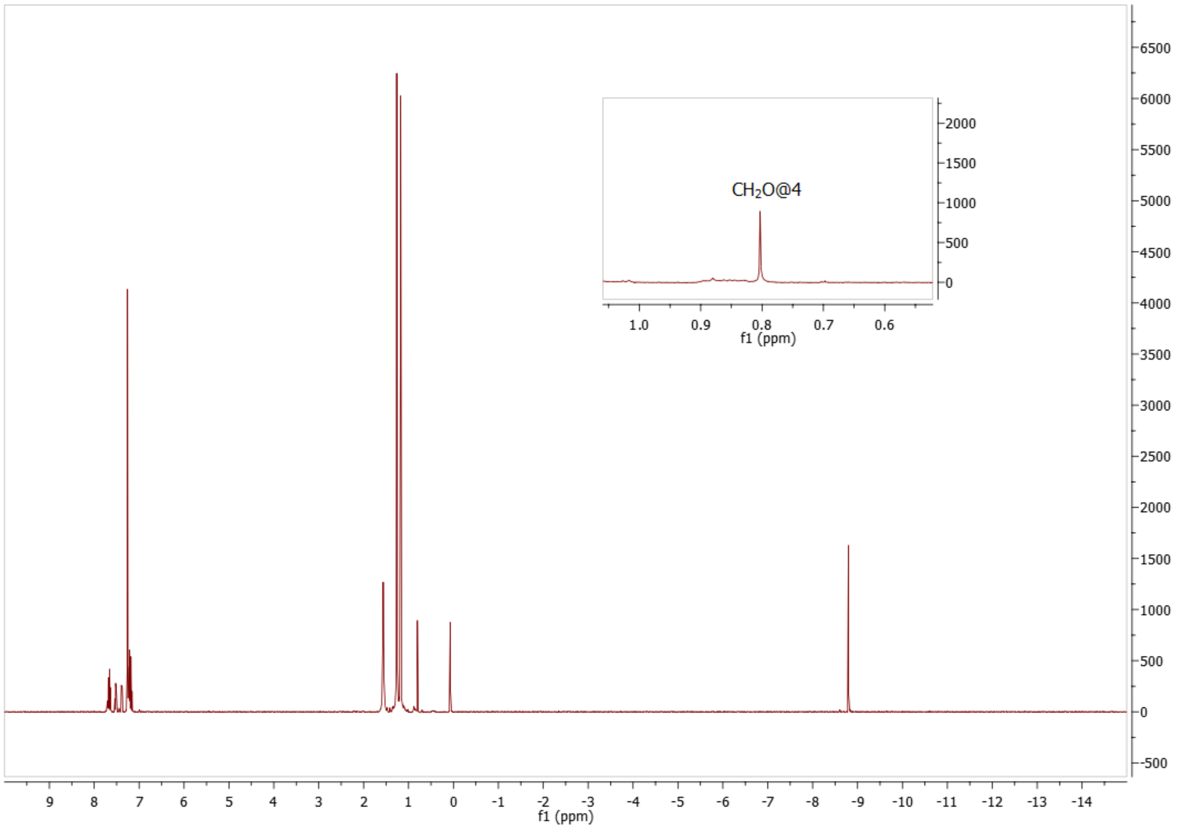

Supplementary Fig. 5. **^{1}H NMR** of mixture of CH_2O@**4** and H_2O@**4** in $CDCl_3$.

^{1}H NMR (400 MHz, *o*-$C_6D_4Cl_2$-*d_4*) δ 7.536 (t, J = 7.8 Hz, 0.35H, pyridyl for CH_2O@**4**), 7.522 (t, J = 7.8 Hz, 0.65H, pyridyl for H_2O@**4**), 7.520 -7.460 (m, 2H, pyridyl for CH_2O@**4** and H_2O@**4**), 7.378 (dd, J = 7.8, 0.8 Hz, 0.35H, pyridyl for CH_2O@**4**), 7.377 (dd, J = 7.8, 0.8 Hz, 0.65H, pyridyl for H_2O@**4**), 7.245 (d, J = 10.0 Hz, 0.35H, alkenyl for CH_2O@**4**), 7.227 (d, J = 10.0 Hz, 0.65H, alkenyl for H_2O@**4**) 7.105 – 7.005 (m, 2H, pyridyl of CH_2O@**4** and H_2O@**4**), 1.271 (*s*, 5.9H, *t*-butyl for H_2O@**4**), 1.268 (*s*, 3.1H, *t*-butyl for CH_2O@**2**), 1.173 (*s*, 3.1H, *t*-butyl for CH_2O@**4**), 1.163 (*s*, 5.9H, *t*-butyl for H_2O@**4**), 0.661 (s, 0.70H, C**H**$_2$O@**4**), -8.947 (s, 1.2H, **H**$_2$O@**4**).

One alkenyl proton is obscured by solvent.

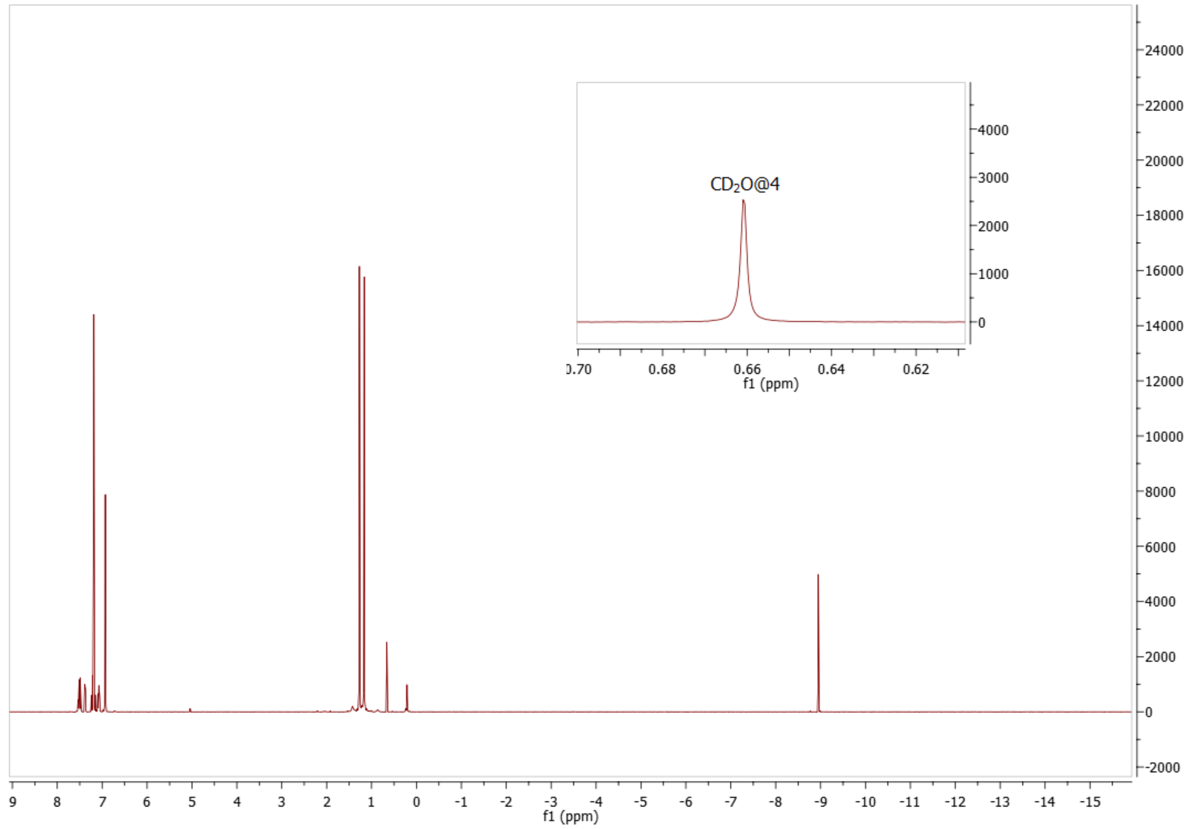

Supplementary Fig. 6. ^{1}**H NMR** of mixture of CH_2O@**4** and H_2O@**4** in *o*-$C_6D_4Cl_2$-*d*$_4$.

CH_2O@4 + H_2O@4 ^{13}C NMR (101 MHz, *o*-$C_6D_4Cl_2$): 199.17 (C), 199.02* (C), 191.47 (C), 190.19* (CH_2), 168.97* (C), 168.92 (C), 168.64* (C), 168.62 (C), 165.52* (C), 165.44 (C), 162.45* (C), 162.44 (C), 154.60 (C), 151.16* (C), 150.09 (C), 149.55* (C), 148.77 (C), 148.75* (C), 148.67* (C), 148.64* (C), 148.46* (C), 147.87 (C), 147.77 (C), 147.66 (C), 147.39 (C), 146.98* (C), 146.67* (C), 146.65* (C), 146.56* (C), 146.52 (C), 146.51 (C), 146.43* (C), 146.35 (C), 146.25 (C), 146.23* (C), 146.21 (C), 146.11* (C), 146.07 (C), 145.94* (C), 145.92* (C), 145.90* (C), 145.88* (C), 145.75 (C), 145.74* (C), 145.67 (C), 145.64* (C), 145.63 (C), 145.59 (C), 145.58 (C), 145.47 (C), 145.45 (C), 145.38 (C), 145.01 (C), 144.84* (C), 144.77* (C), 144.62 (C), 144.60 (C), 144.52 (C), 144.18 (C), 143.86 (C), 143.81 (C), 143.72* (C), 142.77* (C), 142.65 (C), 142.60 (C), 142.08 (C), 141.93* (C), 141.92 (C), 141.87* (C), 141.81 (C), 141.72* (C), 141.65* (C), 141.60 (C), 141.51* (C), 140.99* (C), 140.77 (C), 140.44 (C), 140.40* (C), 140.29 (C), 140.26 (C), 140.17 (C), 140.08 (C), 140.05* (C), 139.90* (C), 139.86* (C), 139.82* (C), 139.60* (C), 139.56 (C), 139.47 (C), 139.39 (C), 139.38 (C), 139.15* (C), 139.04* (C), 138.76* (C), 138.61 (C), 138.44 (C), 138.33 (C), 138.08* (C), 137.58 (C), 137.45* (CH), 137.41 (CH), 137.36 (C), 137.25* (CH), 137.23 (CH), 136.49* (C), 136.48 (C), 136.13* (C), 135.94 (C), 135.76* (C), 135.72 (C), 135.40* (C),

134.88 (C), 134.31* (C), 133.73 (C), 133.27* (C), 132.75 (C), 132.70* (CH), 132.63 (CH), 132.57 (C), 132.19 (C), 131.49* (C), 131.11 (C), 130.01 CHa, 129.93* CH[a], 120.20* (CH), 120.18 (CH), 119.83* (CH), 119.79 (CH), 117.60* (CH), 117.55 (CH), 116.84 (CH), 61.20* (C), 61.15 (C), 55.09* (C), 54.89 (C), 37.70* (C), 37.68 (C), 37.59 (C), 29.94 (CH_3), 29.92 (CH_3). * signals from CH_2O@**4** identified from size and comparison with spectra of pure H_2O@**4**. [a] – from DEPT135 spectra (obscured by *o*-$C_6D_4Cl_2$-d_4 peaks in normal spectra).

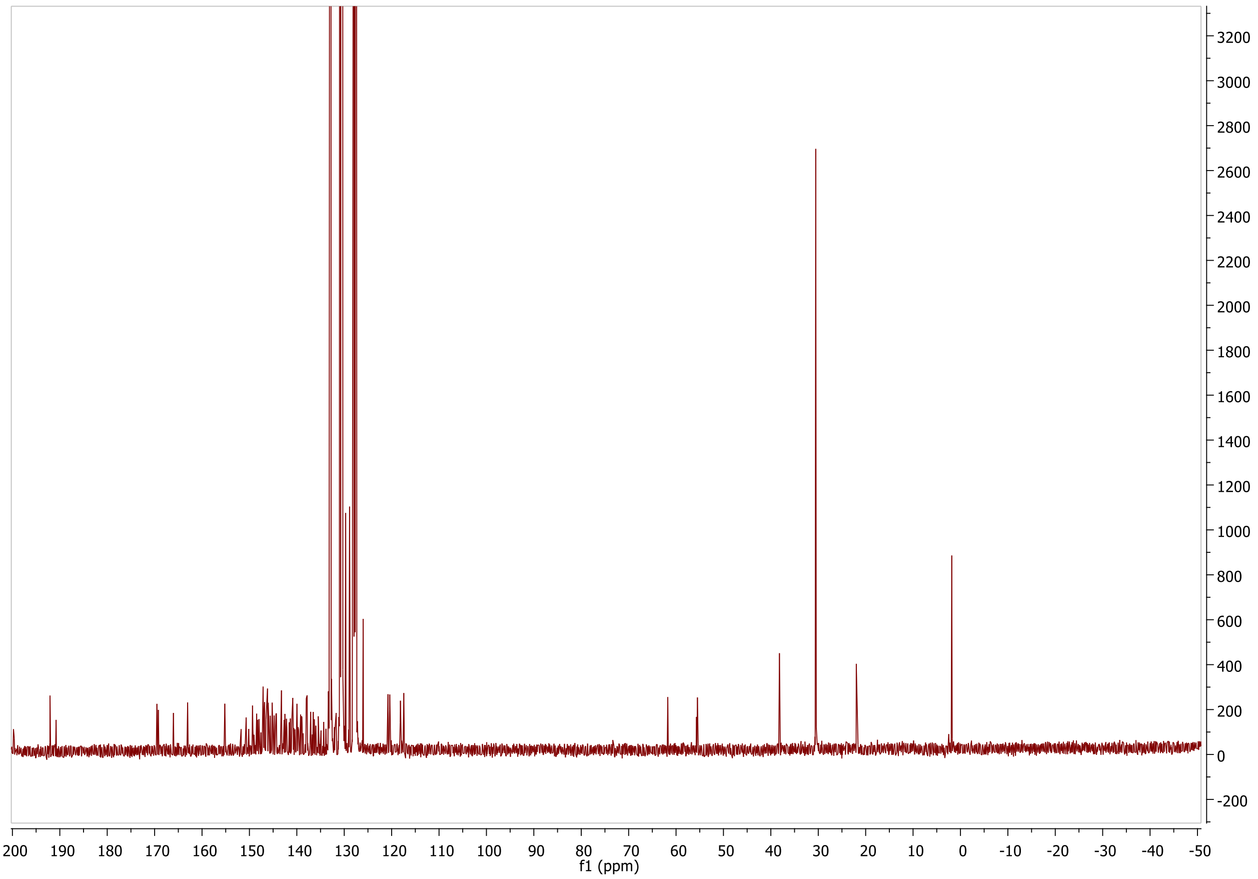

Supplementary Fig. 7. ^{13}C NMR of CH_2O@**4** and H_2O@**4.**

HRMS (ESI+) (*m/z*): Calculated for $[C_{83}H_{29}N_2O_3]^+$ 1101.2173, found 1101.2163 (CH2O@**4**). Calculated for $[C_{82}H_{27}N_2O_2]^+$ 1071.2067, found 1071.2056 (empty **4**). Calculated for $[C_{82}H_{29}N_2O_3]^+$ 1089.2173, found 1089.2168 (H_2O@**4**).

$^{13}CH_2O$@4

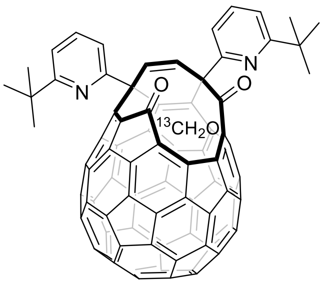

Obtained as mixture of $^{13}CH_2O$@**4** and H_2O@**4** with 8% filling of CH_2O (with a small amount of empty **4** also present).

^{13}C NMR (101 MHz, $CDCl_3$, without proton decoupling) δ 190.47 (t, J = 175.0 Hz, $^{13}CH_2O$@**4**).

HRMS (ESI+) (*m/z*): Calculated for $[^{13}CC_{82}H_{29}N_2O_3]^+$ 1102.2206, found 1102.2177 ($^{13}CH_2O$@**4**). Calculated for $[C_{82}H_{27}N_2O_2]^+$ 1071.2067, found 1071.2037 (empty **4**). Calculated for $[C_{82}H_{29}N_2O_3]^+$ 1089.2173, found 1089.2149 (H_2O@**4**).

CD_2O@4

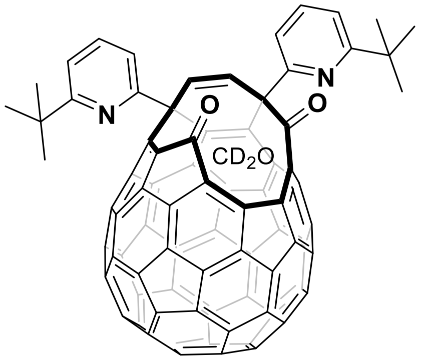

Obtained as a mixture of CD_2O@**4** and H_2O@**4** with 7% CH_2O filling (with a small amount of empty **4** also present)

HRMS (ESI+) (*m/z*): Calculated for $[C_{83}H_{27}D_2N_2O_3]^+$ 1103.2298, found 1103.2271(CD_2O@**4**). Calculated for $[C_{82}H_{27}N_2O_2]^+$ 1071.2067, found 1071.2052 (empty **4**). Calculated for $[C_{82}H_{29}N_2O_3]^+$ 1089.2173, found 1089.2158 (H_2O@**4**).

1.3 $CH_2O@C_{60}$

Intermediate CH_2O@**5** and H_2O@**5** with a ratio of 22:78. ~ 5% empty **5** also present but could not be measured (signals very close to H_2O@**5**).

^{1}H NMR (400 MHz, $CDCl_3$) δ 7.783 (t, J = 7.7 Hz, 0.46H, pyridyl of CH_2O@**5**), 7.777 (t, J = 7.7 Hz, 1.54H, pyridyl H_2O@**5**), 7.725 (dd, J = 7.7, 0.9 Hz, 0.46H, pyridyl of CH_2O@**5**), 7.715 (dd, J = 7.7, 0.9 Hz, 1.54H, pyridyl H_2O@**5**), 7.326 (dd, J = 7.7, 0.9 Hz, 0.46H, pyridyl of CH_2O@**5**), 7.325 (dd, J = 7.7, 0.9 Hz, 1.54H, pyridyl of H_2O@**5**) 6.503 (s, 0.46H, alkenyl of CH_2O@**5**), 6.467 (s, 0.1.54H, alkenyl of H_2O@**5**) 2.922 (s, 0.46H, endohedral CH_2O@**5**), 1.359 (s, 18H, *t*-butyl of CH_2O@**5** + H_2O@**5**), -6.086 (s, 1.43H, endohedral H_2O@**5**).

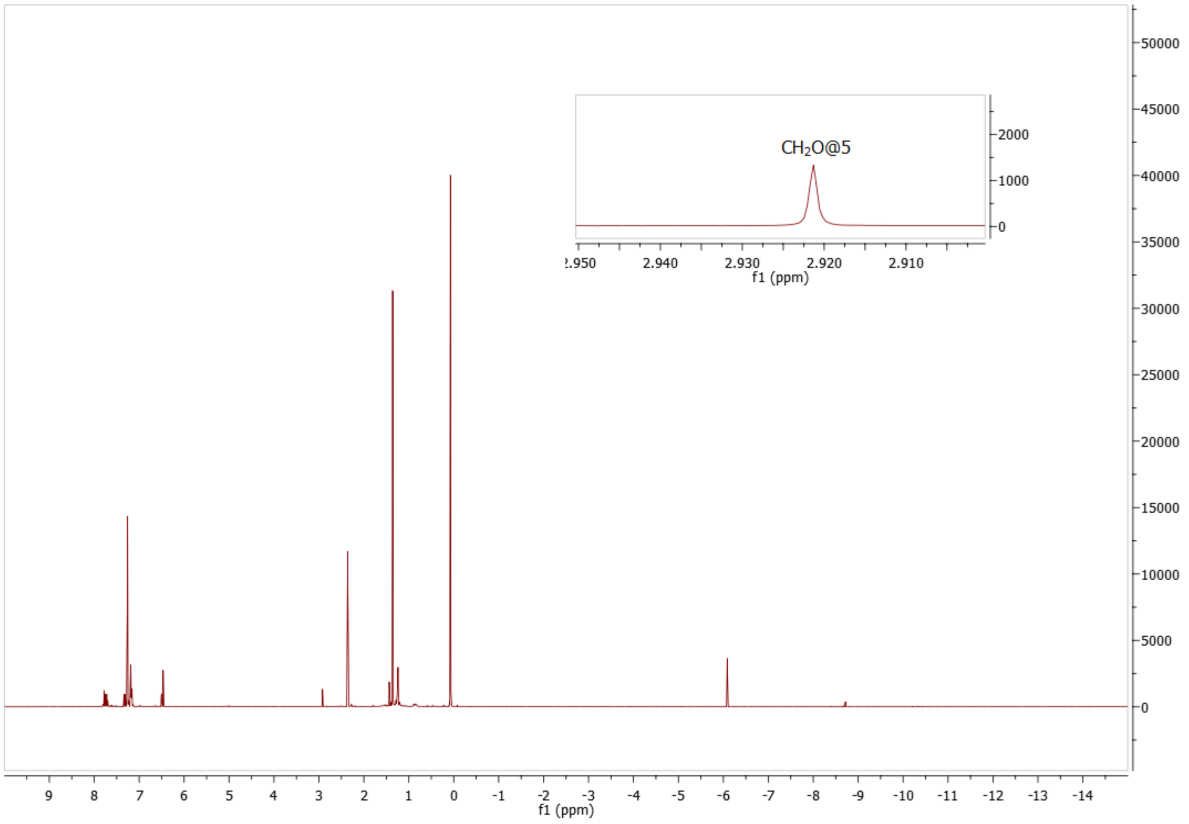

Supplementary Fig. 8. ^{1}H NMR of mixture of CH_2O@**5** and H_2O@**5**.

CH_2O@C_{60}.

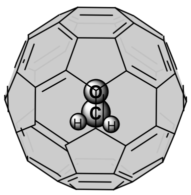

^{1}H NMR (400 MHz, toluene-d_8) δ 3.804 ppm (**H**$_2$CO@C_{60}).

^{13}C NMR (100 MHz, toluene-d_8) δ 144.25 (CH_2O@**C**$_{60}$) (CH_2O carbon not visible)

^{13}C NMR (176 MHz, *o*-$C_6D_4Cl_2$-*d_4*) δ 197.23 (t, J = 173.4 Hz, endohedral **C**H_2O), 143.49 (CH_2O@**C**$_{60}$).

UV (toluene): λ_{max} 409 nm, ε 2344 M^{-1} cm^{-1}. λ_{max} 550 nm, ε 609 M^{-1} cm^{-1}. λ_{max} 602 nm, ε 544 M^{-1} cm^{-1}. λ_{max} 631 nm, ε 276 M^{-1} cm^{-1}.

HRMS (ESI) (*m/z*): Calculated for $[C_{61}H_2O]^{-\bullet}$ 750.0111, found 750.0103.

IR. ν_{max} 2916 (CH stretch of endohedral CH_2O), 1717 (CO stretch of endohedral CH_2O), 1420, 1177, 575 cm^{-1}. For comparison C_{60} ν_{max} 1420, 1177, 575 cm^{-1} (See Supplementary Fig. 11)

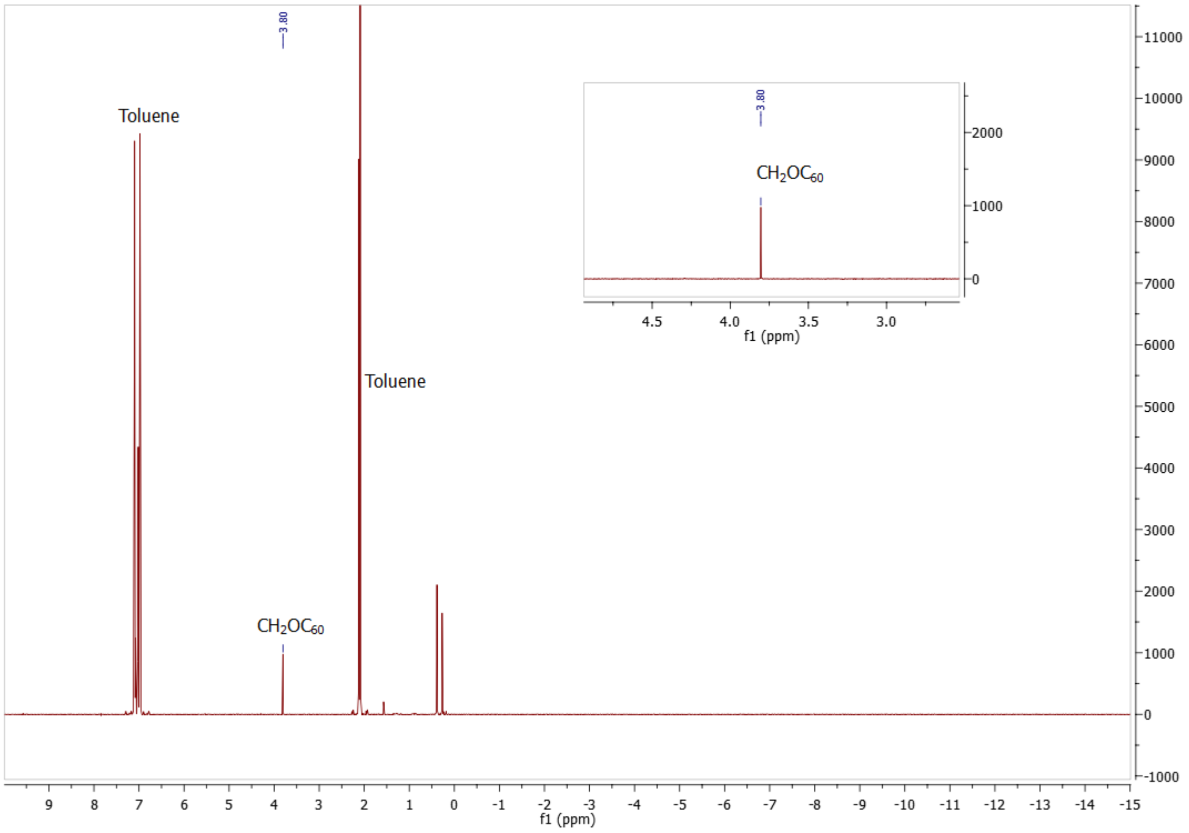

Supplementary Fig. 9. ^{1}H NMR of CH_2O@C_{60}.

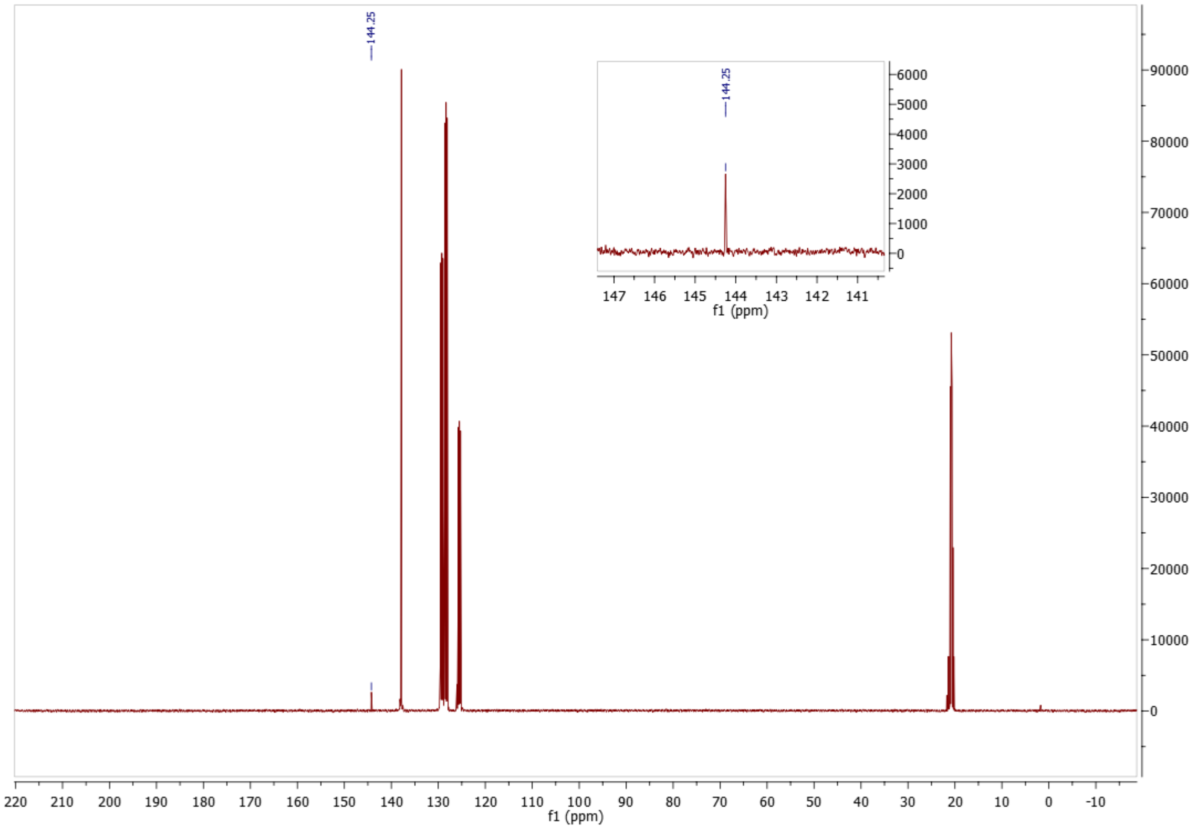

Supplementary Fig. 10. **^{13}C NMR** of $CH_2O@C_{60}$.

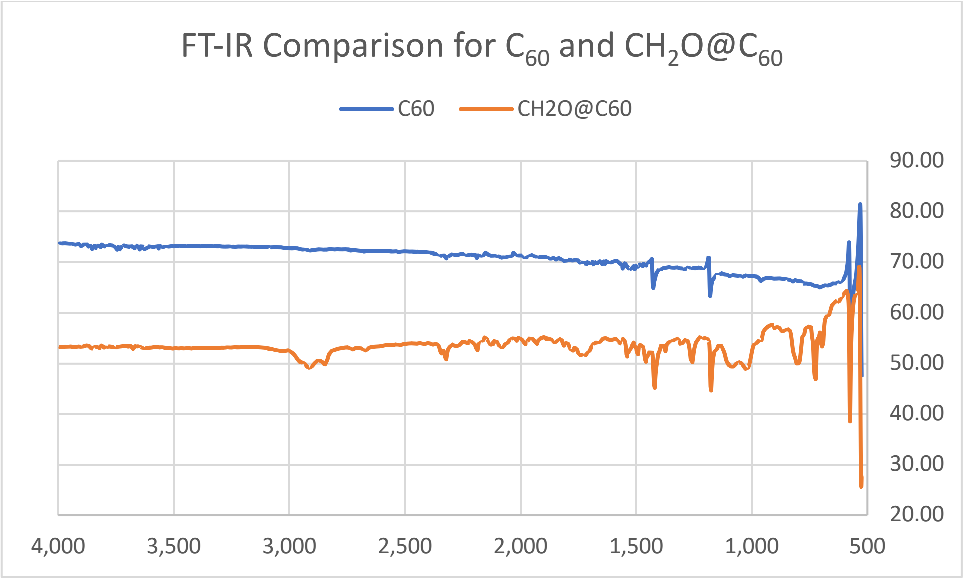

Supplementary Fig. 11. FT-IR comparison for C_{60} and $CH_2O@C_{60}$.

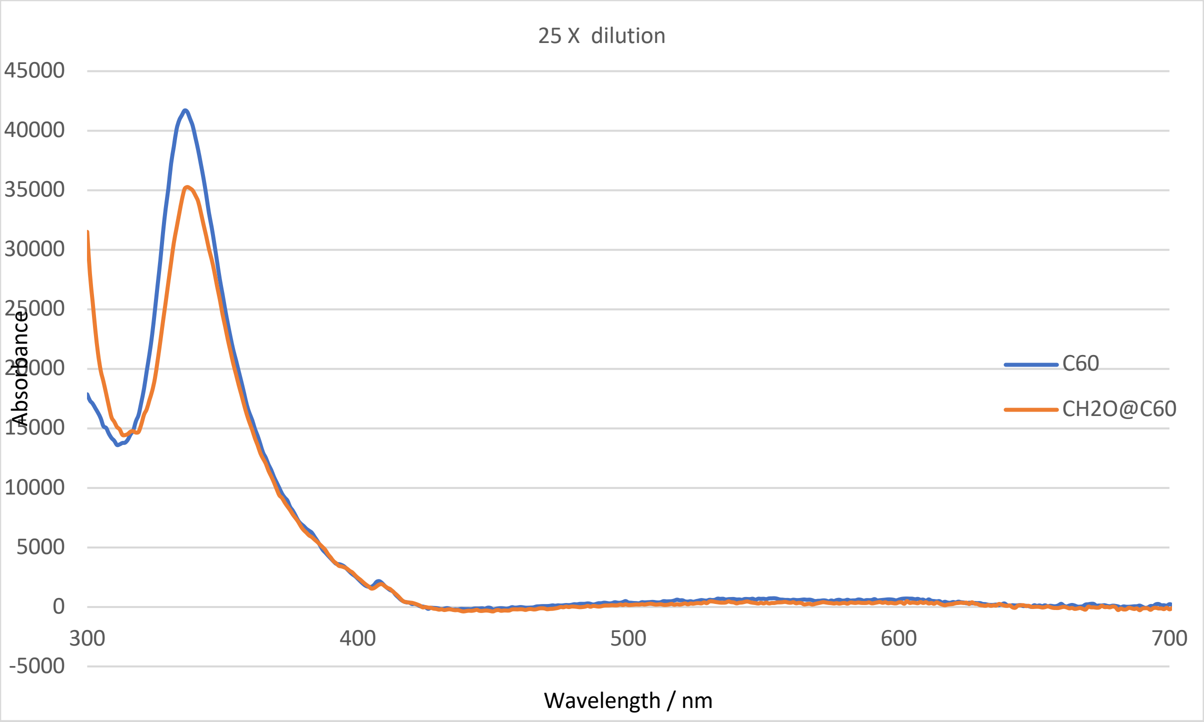

Supplementary Fig. 12. UV-vis spectra of $CH_2O@C_{60}$ and C_{60}. The spectra in the figure was obtained from a solution of the fullerene in toluene ($CH_2O@C_{60}$ approx. 5.33 × 10^{-5} M, C_{60} approx. 5.55 × 10^{-5} M) following serial dilution of a 0.2 mg mL^{-1} solution in toluene. For expansion of long wavelength region see main paper Figure 3a.

$^{13}CH_2O@C_{60}$

^{1}H NMR (700 MHz, toluene-d_8) δ 3.804 ppm (d, J = 173.4 Hz, 1H, **H**$_2{}^{13}CO@C_{60}$)

^{13}C NMR (600 MHz, toluene-d_8. Not proton decoupled) δ 197.93 (t, J = 173.4 Hz, endohedral $CH_2O@C_{60}$), δ 144.25 ($CH_2O@$**C**$_{60}$).

HRMS (ESI) (*m/z*): Calculated for $[^{13}CC_{60}H_2O]^{-\bullet}$ 751.0145, found 751.0133.

$CD_2O@C_{60}$

^{2}H NMR (77 MHz, toluene) δ 3.763.

^{13}C NMR (126 MHz, toluene) δ 143.94. Formaldehyde carbon not observed.

HRMS (ESI) (*m/z*): Calculated for $[C_{61}D_2O]^{-\bullet}$ 752.0237, found 752.0245.

1.4 CH_2O@6

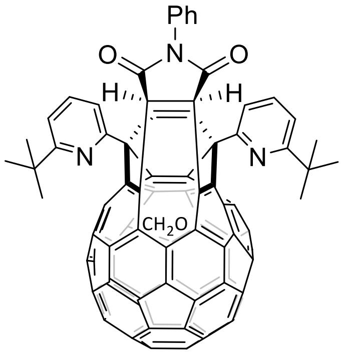

The mixture of CH_2O@**6** and H_2O@**6** + **6** with a ratio of 37:63 (still containing a 13 mol% impurity of N-Ph Maleimide), quoting ^{1}H and ^{13}C signal from CH_2O@**6** and H_2O@**6**.

^{1}H NMR (500 MHz, $CDCl_3$) δ 7.755 (t, J = 7.8 Hz, 0.74H, pyridyl for CH_2O@**6**), 7.749 (t, J = 7.8 Hz, 1.26H, pyridyl for H_2O@**6**), 7.641 (dd, J = 7.7, 0.8 Hz, 0.74H, pyridyl for CH_2O@**6**), 7.631 (dd, J = 7.7, 0.8 Hz, 1.26H, pyridyl for H_2O@**6**), 7.495 – 7.395 (m, 3H, phenyl for CH_2O@**6** and H_2O@**6**), 7.313 (dd, J = 7.9, 0.7 Hz, 0.74H, pyridyl for CH_2O@**6**), 7.308 (dd, J = 7.9 Hz, 0.7 Hz, 1.26H, pyridyl for H_2O@**6**), 7.296 – 7.269 (m, 2H, phenyl for CH_2O@**6** and H_2O@**6**) 7.017 (s, 0.74H, alkenyl for CH_2O@**6**), 6.991 (s, 1.26H, alkenyl for H_2O@**6**), 5.610 (s, 0.74H, alkanyl for CH_2O@**6**), 5.580 (s, 1.26H, alkanyl for H_2O@**6**), 1.333 (s, 7H, *t*-butyl for CH_2O@**6**), 1.331 (s, 11H, *t*-butyl for H_2O@**6**), 1.055 (s, 0.70H, CH_2O@**6**), -8.273 (s, 1.22H, H_2O@**6**).

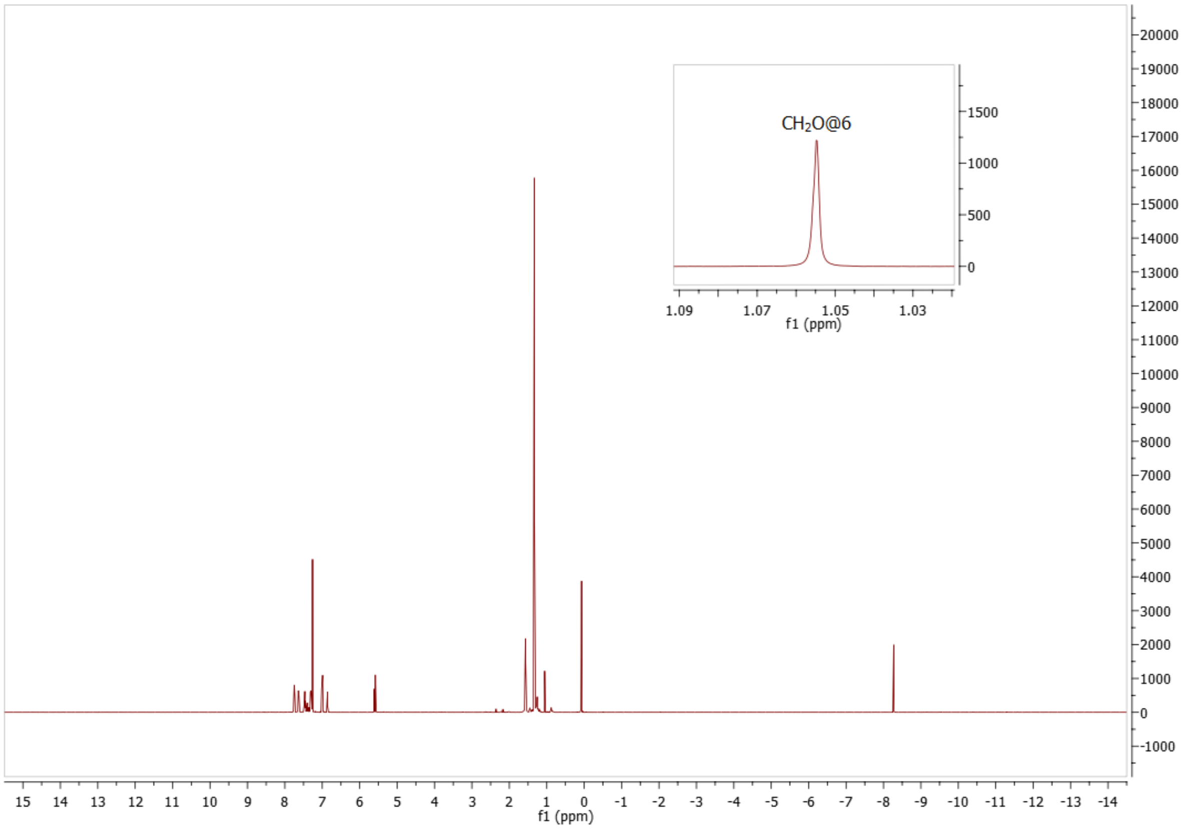

Supplementary Fig. 13. ^{1}H NMR of CH_2O@**6** and H_2O@**6**.

^{13}C NMR (126 MHz, $CDCl_3$) δ 191.52 (CH_2, CH_2O@**6**), 175.82 (C, H_2O@**6**), 175.79 (C, CH_2O@**6**), 169.26 (C, CH_2O@**6**), 169.22 (C, H_2O@**6**), 164.53 (C, CH_2O@b), 164.50 (C, H_2O@**6**), 148.60 (C, CH_2O@**6**), 148.12 (C, CH_2O@**6**), 148.06 (C, CH_2O@**6**), 147.96 (C, H_2O@**6**), 147.76 (C, H_2O@**6**), 147.50 (C, H_2O@**6**), 147.25 (C, CH_2O@**6**), 147.22 (C, H_2O@**6**), 147.21 (C, CH_2O@**6**), 147.20 (C, H_2O@**6**), 147.04 (C, CH_2O@**6**), 146.57 (C, CH_2O@**6**), 146.48 (C, CH_2O@**6**), 146.41 (C, CH_2O@**6**), 146.35 (C, CH_2O@**6**), 146.21 (C, H_2O@**6**), 146.09 (C, H_2O@**6**), 145.98 (C, CH_2O@**6**), 145.94 (C, CH_2O@**6**), 145.88 (C, CH_2O@**6**), 145.70 (C, H_2O@**6**), 145.54 (C, H_2O@**6**), 145.53 (C, H_2O@**6**), 145.44 (C, H_2O@**6**), 145.18 (C, H_2O@**6**), 145.13 (C, H_2O@**6**), 145.08 (C, CH_2O@**6**), 143.12 (C, CH_2O@**6**), 142.75 (C, H_2O@b), 142.65 (C, CH_2O@**6**), 142.44 (C, H_2O@**6**), 141.54 (C, CH_2O@**6**), 141.41 (C, CH_2O@b), 141.19 (C, H_2O@**6**), 141.15 (C, H_2O@**6**), 140.61 (C, CH_2O@**6**), 140.26 (C, CH_2O@**6**), 140.13 (C, CH_2O@**6**), 140.09 (C, H_2O@**6**), 139.93 (C, H_2O@**6**), 139.71 (C, CH_2O@**6**), 139.32 (C, CH_2O@**6**), 138.78 (C, H_2O@**6**), 138.40 (C, H_2O@**6**), 138.23 (C, CH_2O@**6**), 138.19 (C, H_2O@**6**), 137.85 (C, H_2O@**6**), 137.75 (C, H_2O@**6**), 137.47 (C, CH_2O@**6**), 137.35 (CH, CH_2O@**6**), 137.32 (CH, H_2O@**6**), 137.01 (C, CH_2O@**6**), 135.65 (C, CH_2O@**6**), 135.44 (C, H_2O@**6**), 134.87 (C, CH_2O@**6**), 134.60 (C,

H_2O@**6**), 132.26 (C, CH_2O@**6**), 132.11 (C, H_2O@**6**), 131.92 (C, H_2O@b), 131.91 (C, CH_2O@**6**), 131.68 (C, CH_2O@**6**), 131.49 (C, H_2O@**6**), 131.13 (CH, CH_2O@**6**), 131.11 (CH, H_2O@**6**), 130.35 (C, CH_2O@**6**), 129.72 (C, H_2O@**6**), 129.48 (CH, CH_2O@**6**), 129.47 (CH, H_2O@**6**), 129.23 (CH, CH_2O@**6**), 129.21 (CH, H_2O@**6**), 126.67 (CH, H_2O@**6**+CH_2O@**6**), 120.31 (CH, CH_2O@**6**), 120.26 (CH, H_2O@**6**), 117.46 (CH, CH_2O@**6**), 117.43 (CH, H_2O@**6**), 58.28 (C, CH_2O@**6**), 58.06 (C, H_2O@**6**), 54.00 (CH, H_2O@**6**+CH_2O@**6**), 37.99 (C, CH_2O@**6**), 37.98 (C, H_2O@**6**), 30.16 (CH_3, H_2O@**6**+CH_2O@**6**). Spectra assigned to CH_2O@**6** or H_2O@**6** using the relative sizes of peaks, the reported spectra of H_2O@**6**,[5] and HSQC C-H correlation spectra.

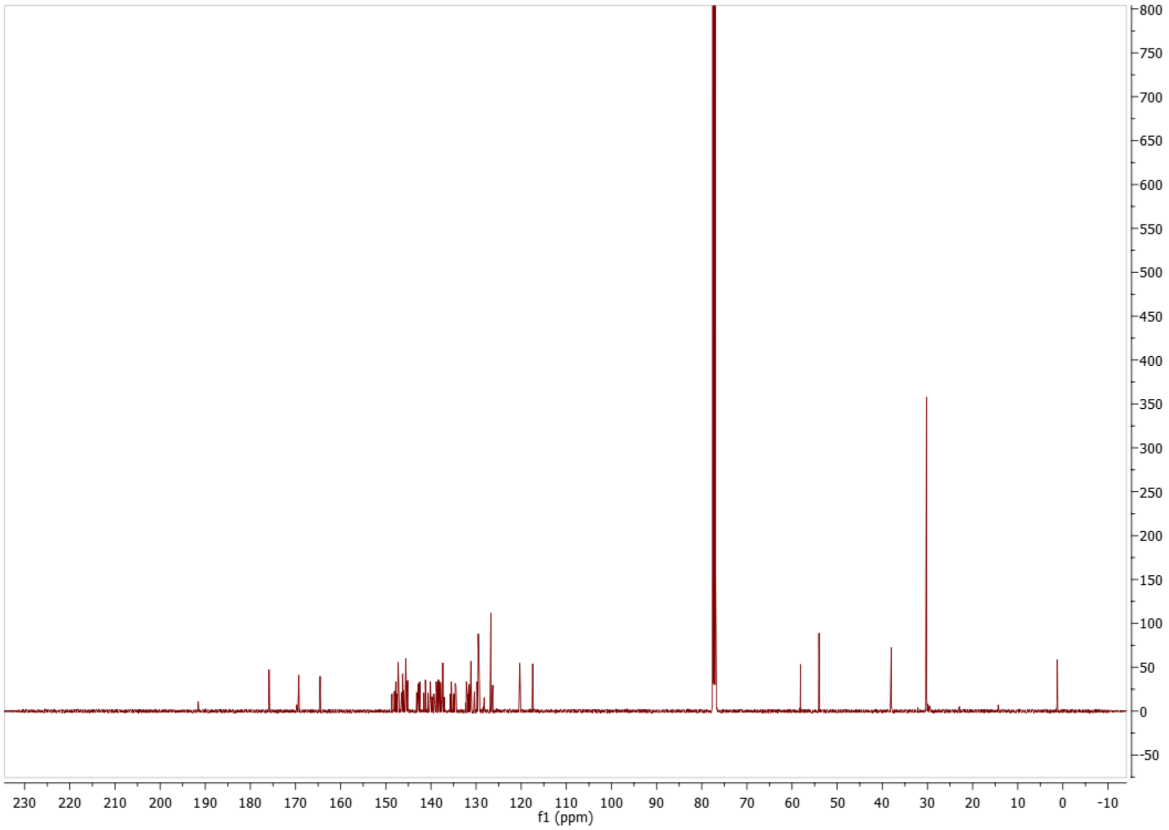

Supplementary Fig. 14. ^{13}C NMR of CH_2O@**6** and H_2O@**6**.

HRMS (ESI+) (*m/z*): Calculated for $[C_{93}H_{36}N_3O_3]^+$ 1242.2751, found 1242.2727(CH_2O@**6**). Calculated for $[C_{92}H_{34}N_3O_2]^+$ 1212.2646, found 1212.2610 (empty **6**). Calculated for $[C_{92}H_{34}N_3O_3]^+$ 1230.2751, found 1230.2726 (H_2O@**6**).

1.5 CO_2@Sulfoxide

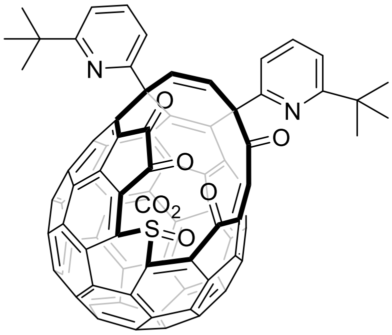

The mixture of CO_2@**2** and H_2O@**2** with a ratio of 90:10. Only peaks from CO_2@**2** are listed. ^{1}H NMR (400 MHz, $CDCl_3$) δ 7.609 (t, J = 7.9 Hz, 1H, pyridyl), 7.585 (t, J = 7.9 Hz, 1H, pyridyl), 7.312 (dd, J = 7.9, 0.8 Hz, 1H, pyridyl). 7.278-7.145 (m, 4H, pyridyl and alkenyl protons, region mainly obscured by the toluene), 6.805 (d, J = 10.3 Hz, 1H), 1.182 (*s*, 9H, *t*-butyl).

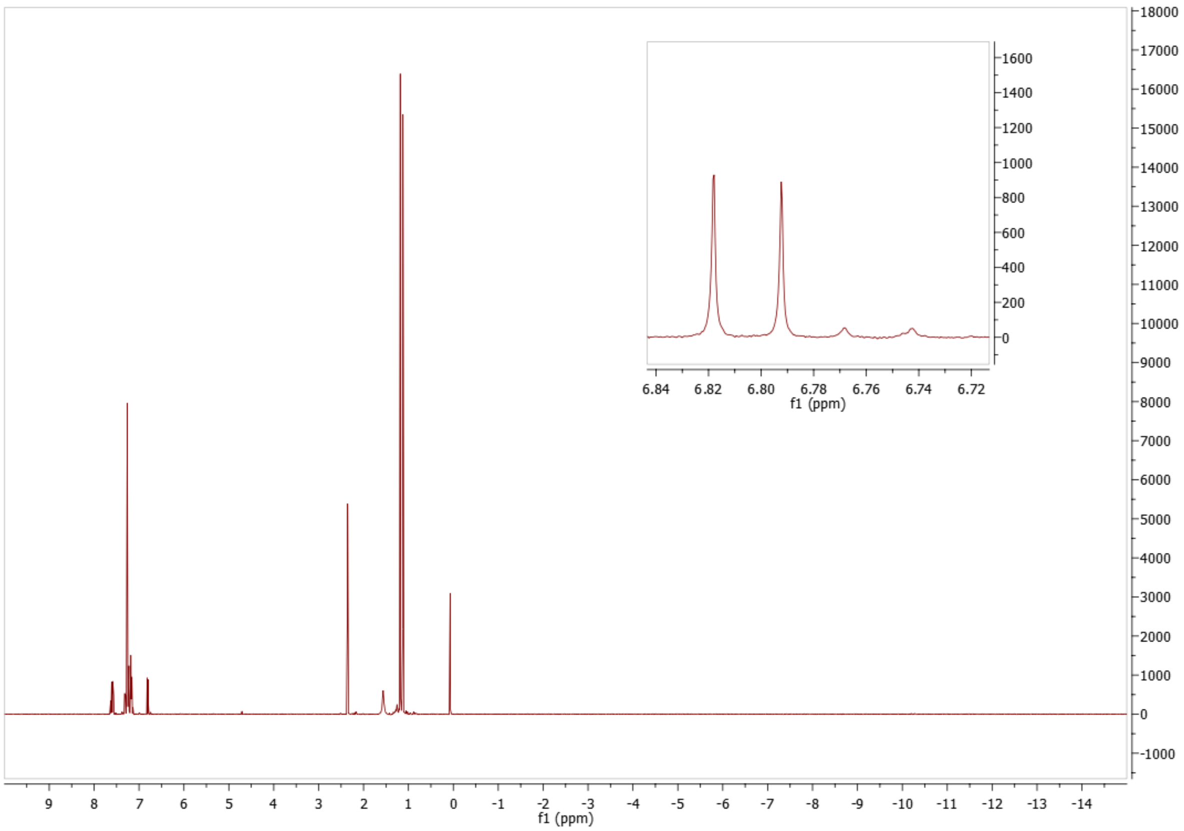

Supplementary Fig. 15. ^{1}H NMR of CO_2@**2** and H_2O@**2**.

^{13}C NMR (101 MHz, $CDCl_3$) δ 190.66(C), 185.08(C), 184.45(C), 180.88(C), 169.05(C), 168.72(C), 163.34(C), 162.08(C), 157.17(C), 155.77(C), 151.62(C), 151.42(C), 151.20(C),

151.18(C), 151.00(C), 150.84(C), 150.78(C), 150.68(C), 150.65(C), 150.44(C), 150.35(C), 149.81(C), 149.65(C), 148.12(C), 147.89(C), 147.56(C), 147.12(C), 145.18(C), 145.06(C), 144.80(C), 144.68(C), 144.62(C), 144.43(C), 143.94(C), 143.78(C), 143.65(C), 143.46(C), 143.26(C), 143.16(C), 141.89(C), 141.83(C), 140.85(C), 140.71(C), 140.57(C), 139.39(C), 139.13(C), 138.76(C), 138.08(), 137.94(C), 137.74(CH), 137.50(C), 137.43(CH), 137.15(C), 136.98(C), 136.87(C), 136.63(C), 136.37(C), 136.34(C), 136.12(C), 134.43(C), 133.91(C), 131.81(C), 131.37(C), 129.88(C), 128.10(C), 126.33(C), 126.01(C), 125.91(CH), 120.38(CH), 119.77(CH), 117.86(CH), 117.67(CH), 114.21 (CH), 59.49(C), 55.22(C), 37.94(C), 37.91(C), 31.15 (C), 30.09 ($6xCH_3$). Only signals from CO_2@**2** reported.

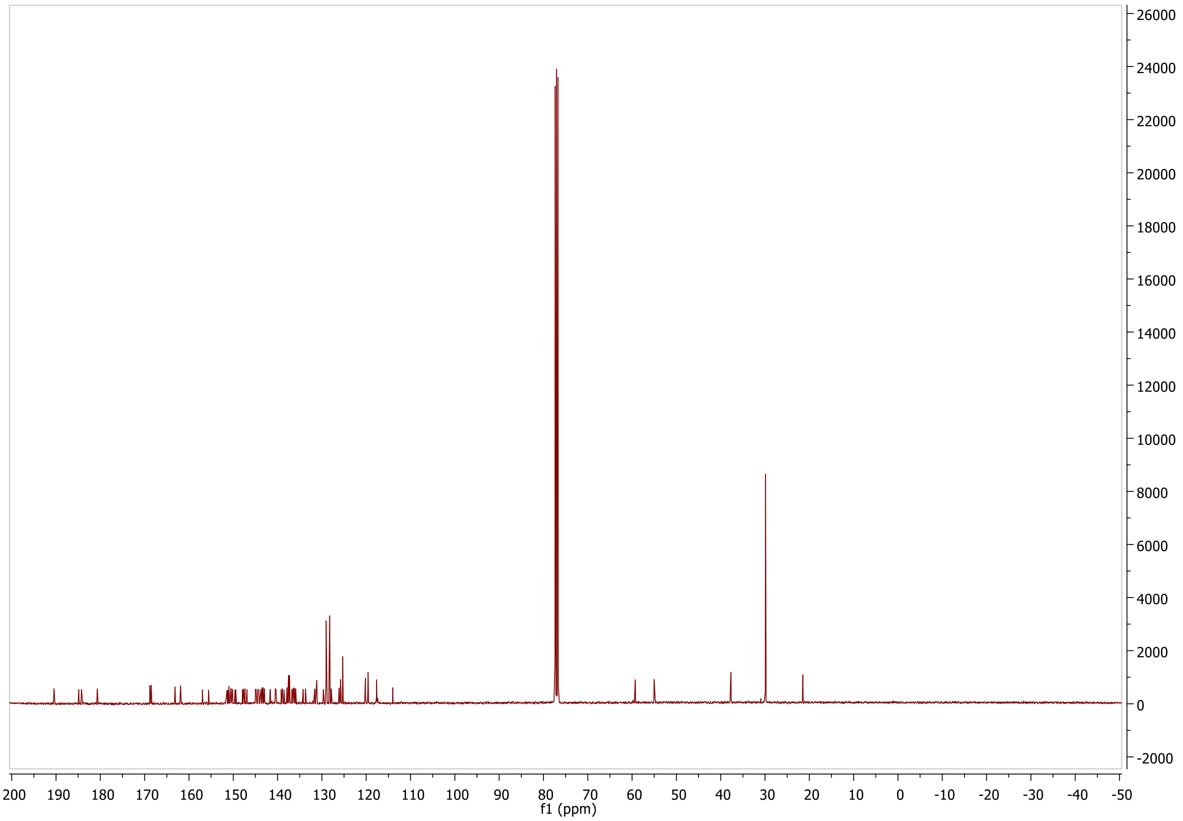

Supplementary Fig. 16. ^{13}C NMR of CO_2@**2**.

HRMS (ESI+) (*m/z*): Calculated for $[C_{83}H_{27}N_2O_7S]^+$ 1195.1553, found 1195.1550(CO_2@**2**). Calculated for $[C_{82}H_{27}N_2O_5S]^+$ 1151.1635, found 1151.1651 (empty).

2. Kinetics of loss of CH_2O from CH_2O@1 and CH_2O@2 and of CO_2 from CO_2@2,

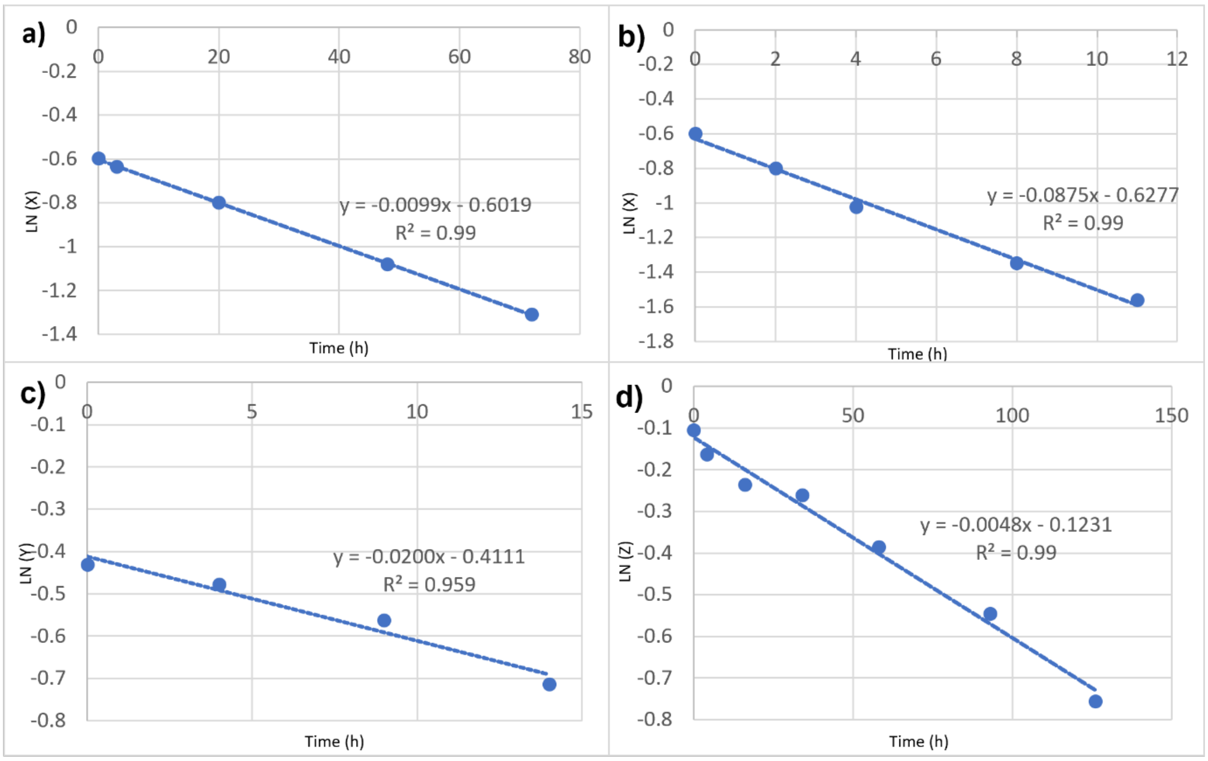

X = ([CH_2O@1]\[CH_2O@1+H_2O@1 +1]) Y = ([CH_2O@2]\[CH_2O@2+H_2O@2 + 2]) Z = ([CO_2@2\[CO_2@2+H_2O@2+2])

Supplementary Fig. 17. 1st order plots for loss of: (a) CH_2O from CH_2O@**1** at 25 °C and (b) 40 °C; (c) CH_2O from CH_2O@**2** at 55°C; (d) loss of CO_2 from CO_2@**2** at 55 °C.

Supplementary Table 1. Loss of CH_2O from CH_2O@**1**

25 °C		40 °C	
**Time**	**Filling Factor**	**Time**	**Filling Factor**
0	0.55	0	0.55
3	0.53	2	0.45
20	0.45	4	0.36
48	0.34	8	0.26
72	0.27	11	0.21
Half-life = 70h		Half-life = 8h	

Supplementary Table 2. Loss of CH_2O from CH_2O@2 at 55 °C.

55 °C	
**Time**	**Filling Factor**
0	0.65
4	0.62
9	0.57
14	0.49
Half-life = 34.6 h	

Supplementary Table 3. Loss of CO_2 from CO_2@2 at 55 °C

**55 °C**	
**Time**	**Filling Factor**
0	0.90
4	0.85
16	0.79
34	0.77
58	0.69
93	0.58
126	0.47
Half-life = 144 h	

3. Voltametry of CH_2O@C_{60}.

For DPV see Fig. 3b in main paper.

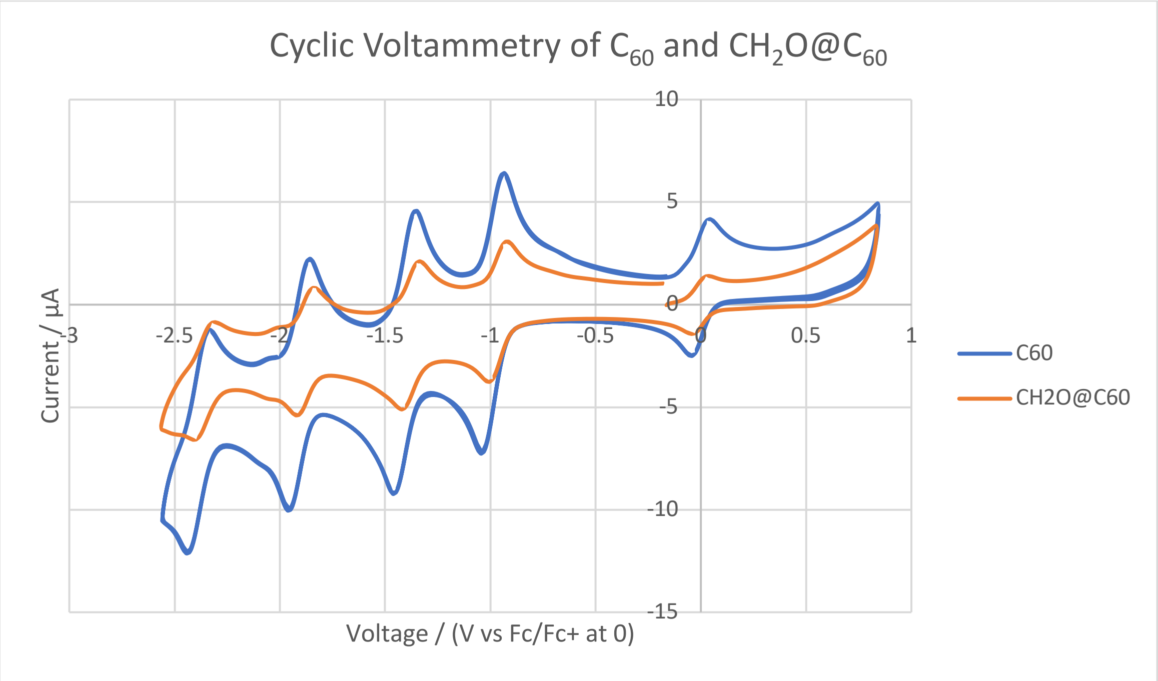

Supplementary Fig. 18. Cyclic Voltammetry of C_{60} and CH_2O@C_{60}.

Supplementary Table 4. Reduction potential of C_{60} and CH_2O@C_{60} reported relative to the Fc/Fc^+ couple at 0V.

	CV		DPV	
Reduction	C_{60}	CH_2O@C_{60}	C_{60}	CH_2O@C_{60}
1st	-0.984	-0.953	-0.982	-0.952
2nd	-1.398	-1.372	-1.405	-1.375
3rd	-1.902	-1.874	-1.898	-1.868
4th	-2.380	-2.351	-2.377	-2.346

4. Density Functional Theory Calculations

Cartesian coordinates are in a separate file.

Model structures **1a** and **2a** were used to represent compounds **1** and **2**, in which the 6-tert-butylpyridyl groups were replaced by methyl substituents. Geometry optimisation and frequency calculations used the cc-pVDZ basis set. Electronic energies and counterpoise correction for Basis Set Superposition Error used the cc-pVTZ basis set.

Supplementary Table 5. DFT Energies (Hartree) for calculated species

	B3LYP			M06-2X		
	E[a]	BSSE[b]	G[c]	E[a]	BSSE[b]	G[c]
H_2O	-76.4603		0.0034	-76.4253		0.0040
CH_2O	-114.5510		0.0053	-114.4966		0.0060
CO_2	-188.6622		-0.0091	-188.5916		-0.00867
**1a**	-3219.9584		0.4590	-3218.6794		0.4659
**2a**	-3295.1660		0.4607	-3293.8558		0.4677
H_2O@**1a**	-3296.4438	0.0060	0.4783	-3295.1309	0.0048	0.4867
TS **1a**+H_2O ⇌ H_2O@**1a**	-3296.4304	0.0066	0.4822	-3295.1197	0.0052	0.4894
CH_2O@**1a**	-3334.5379	0.0032	0.4849	-3333.2060	0.0031	0.4925
TS **1a**+CH_2O ⇌ CH_2O@**1a**	-3334.5045	0.0034	0.4883	-3333.1731	0.0032	0.4957
CH_2O@**2a**	-3409.7454	0.0033	0.4859	-3408.3825	0.0032	0.4947
TS **2a**+CH_2O ⇌ CH_2O@**2a**	-3409.7070	0.0034	0.4894	-3408.3449	0.0034	0.4982
CO_2@**2a**	-3483.8538	0.0032	0.4699	-3482.4743	0.0033	0.4766
TS **2a**+CO_2 ⇌ CH_2O@**2a**	-3483.8089	0.0031	0.4722	-3482.4317	0.0030	0.4798

a. Electronic energy. b. correction for basis set superposition error. c. Thermal correction to give free energy at STP.

Supplementary Table 6. Calculated free energies of binding, entry and exit of the endohedral species in kJ mol^{-1}.

	B3LYP			M06-2X		
	G (bind)	G^{act} (entry)	G^{act} (exit)	G (bind)	G^{act} (entry)	G^{act} (exit)
**1a**+H_2O ⇌ H_2O@**1a**	-8.5	38.2	46.8	-11.9	25.6	37.5
**1a**+CH_2O ⇌ CH_2O@**1a**	-12.4	84.5	96.9	-16.8	78.0	94.9
**2a**+CH_2O ⇌ CH_2O@**2a**	-13.8	96.5	110.3	-15.9	92.5	108.4
**2a**+CO_2 ⇌ CO_2@**2a**	-10.6	113.2	123.8	-16.0	103.7	119.7

The activation energy and binding energy (ΔG at 298K) for entry of CH_2O into a model for **1** in which the pyridyl tert-butyl groups are replaced by hydrogens using M06-2X / 6-31G* has been reported[1] as 16.6 kCal/mol (69.5 kJ/mol) and -9.9 kCal/mol (-41.4 kJ/mol) without

counterpoise correction for the basis set superposition error[2] which makes a significant difference (our M06-2X/cc-pVTZ//cc-pVDZ values are 69.6 and -25.0 kJ/mol without correction). Our calculations at the M06-2X/6-31G* level give ΔG for entry and binding of 65.8 and -35.0 kJ/mol (15.7 and -8.4 kCal/mol) respectively without counterpoise correction in good agreement with the published values above, but these become and 90.4 and -11.8 kJ/mol (21.6 and -2.8 kCal/mol) with correction illustrating its impact, particularly with small basis sets.

4.2. Calculated Properties of $CH_2O@C_{60}$.

Cartesian coordinates are in a separate file.

4.2.1 Minimised structures of $CH_2O@C_{60}$ and C_{60}.

Two very close minimum energy structures for $CH_2O@C_{60}$ were identified at the cc-pVTZ/tight level. One in which the CH_2O C-O axis was aligned with the centre of the bond between a 5 and 6 member ring (formally a single bond) denoted '$CH_2O@C_{60}$ 56 iso' and one aligned with the centre of the bond between two six member rings (formally a double bond) denoted '$CH_2O@C_{60}$ 66 iso' (Supplementary Fig. 19). The later was 0.1 kJ mol^{-1} higher in energy – confirmed by single point energy calculations at the cc-pVQZ level and not changed in either case by inclusion of Basis Set Superposition Error correction (using counterpoise calculations).[2] As they are so close in energy results for both conformers are given below.

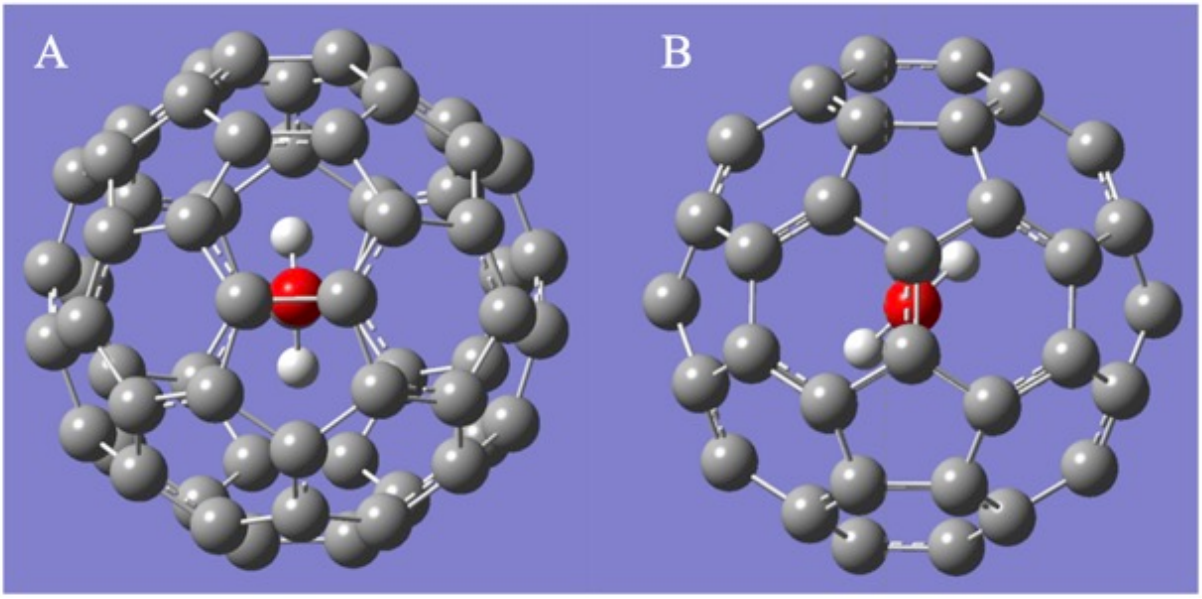

Supplementary Fig. 19. Minimum energy conformers of $CH_2O@C_{60}$**.** **A** – $CH_2O@C_{60}$ 56 iso. **B-** $CH_2O@C_{60}$ 66 iso.

Supplementary Table 7. Diameters of $CH_2O@C_{60}$ and C_{60} in Å.

$CH_2O@C_{60}$ 56 iso				$CH_2O@C_{60}$ 66 iso				C_{60}		
C36	C44	7.0768	c	C1	C52	7.1212	a	C26	C50	7.0789
C32	C21	7.0792		C42	C7	7.1204	a	C27	C49	7.0787
C33	C20	7.0898		C10	C40	7.0963		C51	C8	7.0789
C57	C14	7.0778		C9	C41	7.0893		C2	C53	7.0791
C58	C45	7.0765	c	C8	C51	7.1041		C3	C56	7.0789
C54	C11	7.0614		C2	C53	7.1173		C46	C60	7.0791
C55	C15	7.0715		C50	C26	7.0676		C47	C59	7.0790
C24	C23	7.0811		C48	C35	7.0837		C1	C52	7.0791
C25	C22	7.0694	b	C49	C27	7.0669		C41	C9	7.0789
C34	C12	7.0591	b	C57	C14	7.0648		C35	C48	7.0789
C51	C8	7.0718		C33	C20	7.0642		C54	C11	7.0790
C35	C48	7.0598		C32	C21	7.0599	b	C55	C15	7.0788
C27	C49	7.0620		C36	C44	7.0604	b	C24	C23	7.0790
C26	C50	7.0738		C58	C45	7.0650		C25	C22	7.0789
C41	C9	7.0850		C43	C4	7.0908		C34	C12	7.0788
C39	C6	7.1156	a	C38	C5	7.1050		C16	C31	7.0786
C42	C7	7.1181	a	C31	C16	7.0841		C5	C38	7.0786
C40	C10	7.1102		C30	C17	7.0672		C4	C43	7.0788
C29	C18	7.1094		C37	C13	7.0685		C13	C37	7.0789
C28	C19	7.1063		C23	C24	7.0793		C17	C30	7.0788
C37	C13	7.0845		C15	C55	7.0715		C58	C45	7.0790
C43	C4	7.1084		C11	C54	7.0800		C36	C44	7.0788
C38	C5	7.1050		C12	C34	7.0796	c	C32	C21	7.0791
C31	C16	7.0785		C22	C25	7.0794	c	C33	C20	7.0791
C30	C17	7.0696		C60	C46	7.0823		C57	C14	7.0789
C1	C52	7.1077		C56	C3	7.0952		C40	C10	7.0788
C2	C53	7.0806		C59	C47	7.0980		C29	C18	7.0788
C3	C56	7.0703		C39	C6	7.1167		C28	C19	7.0788
C46	C60	7.0848		C28	C19	7.0938		C39	C6	7.0789
C47	C59	7.1092		C29	C18	7.0806		C42	C7	7.0789
Average:		7.08511		Average:		7.08510		Average:		7.07889

a Along CH_2O CO-axis. b Perpendicular to CH_2O plane. c In plane of CH_2O

Supplementary Table 8. Calculated diameters of $CH_2O@C_{60}$ and C_{60} cages.

	$CH_2O@C_{60}$ 56 iso			$CH_2O@C_{60}$ 66 iso		
		Difference / pm			Difference / pm	
Diameters	Diameter/Å	from average	from C_{60}	Diameter/Å	from average	from C60
Average	7.08511		0.621	7.08510		0.621
Along CO-axis	7.1169	3.17	3.80	7.1208	3.57	4.19
Perpendicular to CH_2O plane	7.0643	-2.09	-1.46	7.0602	-2.50	-1.87
In plane of CH_2O	7.0767	-0.85	-0.22	7.0795	-0.56	0.06

4.2.2 Molecular orbital energies of $CH_2O@C_{60}$ and C_{60}.

Supplementary Table 9. Calculated Molecular Orbital Energies of $CH_2O@C_{60}$ and C_{60}.

	C_{60}		$CH_2O@C_{60}$ 56 iso			$CH_2O@C_{60}$ 66 iso		
	orbital energies		orbital energies		difference from C_{60}	orbital energies		difference from C_{60}
	Ha	eV	Ha	eV	meV	Ha	eV	meV
	-0.08692	-2.3652	-0.0882	-2.4000	-34.8	-0.08808	-2.3968	-31.6
	-0.13211	-3.5949	-0.13267	-3.6101	-15.2	-0.13275	-3.6123	-17.4
	-0.13211	-3.5949	-0.1328	-3.6137	-18.8	-0.13278	-3.6131	-18.2
LUMO	-0.13211	-3.5949	-0.13339	-3.6297	-34.8	-0.13332	-3.6278	-32.9
HOMO	-0.23308	-6.3424	-0.23295	-6.3389	3.5	-0.23303	-6.3411	1.4
	-0.23308	-6.3424	-0.23338	-6.3506	-8.2	-0.23332	-6.3489	-6.5
	-0.23308	-6.3424	-0.23349	-6.3536	-11.2	-0.23344	-6.3522	-9.8
	-0.23308	-6.3424	-0.23382	-6.3626	-20.1	-0.23367	-6.3585	-16.1
	-0.23308	-6.3424	-0.23436	-6.3772	-34.8	-0.23457	-6.3830	-40.5
	-0.28262	-7.6905	-0.28158	-7.6622	28.3	-0.2814	-7.6573	33.2
HOMO-LUMO gap	0.10097	2.7475	0.09956	2.709	-38.4	0.09971	2.7132	-34.3

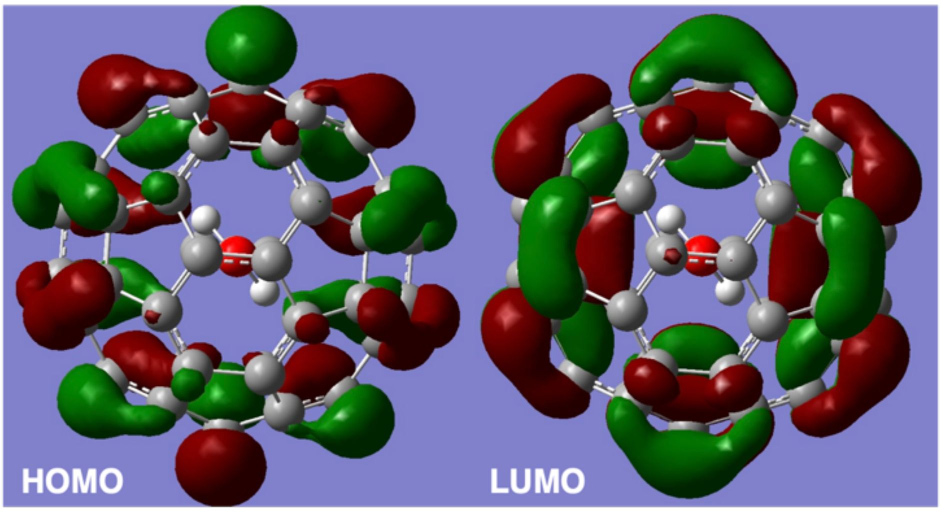

Supplementary Fig. 20. HOMO and LUMO of $CH_2O@C_{60}$ 66 iso. (for $CH_2O@C_{60}$ 56 iso see main paper)

4.2.3 Translational modes of $CH_2O@C_{60}$.

Supplementary Table 10. Calculated translational modes of CH_2O in $CH_2O@C_{60}$ (B3LYP-D3/cc-pVTZ)

		$CH_2O@C_{60}$ 56 iso		$CH_2O@C_{60}$ 66 iso	
		E/cm^{-1}	IR intensity	E/cm^{-1}	IR intensity
Trans 1	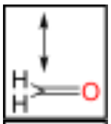	175.3	0.03	174.5	0.03
Trans 2	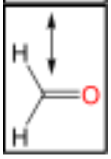	202.52	0.05	202.1	0.03
Trans 3	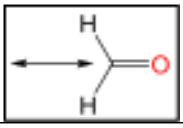	233.04	0.12	233.6	0.12

4.2.4 Calculation of excited state energies.

Using single point TD-DFT calculations for singlet states at ground state geometries using B3LYP-D3/cc-pVTZ with superfine integration grid.

For $CH_2O@C_{60}$ 56 iso the 1st excited state energy E(TD-HF/TD-KS) = -2401.61773452 Ha. Ground state energy is -2401.69391 Ha so excitation energy is 0.075656 Ha = 2.0587 eV which corresponds to absorption at 602.24 nm.

For $CH_2O@C_{60}$ 66 iso the 1st excited state energy E(TD-HF/TD-KS) = -2401.61754357 Ha. Ground state energy is -2401.69336 Ha so excitation energy is 0.07582 Ha = 2.0631 eV which corresponds to absorption at 600.97 nm.

For C_{60} 1st excited state energy E(TD-HF/TD-KS) = -2287.05073288 Ha. Ground state energy = -2287.127262 Ha so excitation energy is 0.07653 Ha = 2.0825 eV which corresponds to absorption at 595.37 nm. Thus $CH_2O@C_{60}$ 56 iso excitation energy decreases by 23.8 meV corresponding to a 6.9 nm change in absorption wavelength. For $CH_2O@C_{60}$ 66 iso excitation energy decreases by 19.4 meV corresponding to a 3.6 nm change in absorption wavelength. The significant difference is probably related to proximity of the carbonyl oxygen to a formal single bond in $CH_2O@C_{60}$ 56 iso, and formal double bond in $CH_2O@C_{60}$ 66 iso.

In practice fullerenes have many very close excited states – the 10 lowest from $CH_2O@C_{60}$ and C_{60} are given in the table below.

Supplementary Table 11. Singlet excited state energies for $CH_2O@C_{60}$ and C_{60}.

	$CH_2O@C_{60}$ 56 iso		$CH_2O@C_{60}$ 66 iso		C_{60}	
State	Excitation energy/ eV	l / nm	Excitation energy / eV	l / nm	Excitation energy / eV	l / nm
1	2.0587	602.24	2.0631	600.97	2.0825	595.37
2	2.0615	601.43	2.0652	600.36	2.0825	595.36
3	2.0731	598.05	2.0692	599.19	2.0825	595.35
4	2.0763	597.13	2.0735	597.93	2.0826	595.34
5	2.0785	596.52	2.0774	596.83	2.0952	591.75
6	2.085	594.66	2.0824	595.39	2.0953	591.74
7	2.0871	594.04	2.0888	593.58	2.0953	591.72
8	2.0874	593.96	2.0931	592.35	2.096	591.53
9	2.1043	589.19	2.0937	592.18	2.096	591.52
10	2.1127	586.85	2.1202	584.78	2.0961	591.51

4.2.5 NMR calculations on $CH_2O@C_{60}$ and C_{60}.

Isotropic ^{13}C NMR shifts of $CH_2O@C_{60}$ and C_{60} were calculated using the GAIO method using B3LYP with cc-pVTZ basis set and superfine integration grid.

Supplementary Table 12. Calculated ^{13}C NMR shifts for C_{60} and $CH_2O@C_{60}$.

C60	$CH_2O@C_{60}$ 56 iso	$CH_2O@C_{60}$ 66 iso	C_{60}[a]	C_{60}[b]	$CH_2O@C_{60}$[c]
33.318	29.535	27.993	31.890	31.465	34.102
33.354	31.875	28.828	33.087	32.165	34.246
33.329	32.742	31.486	33.794	33.613	33.690
33.316	30.114	31.375	32.373	32.637	33.498
33.350	29.822	30.853	32.027	32.555	34.070
33.365	28.585	28.923	31.997	32.168	33.297
33.316	28.330	28.069	31.865	31.473	33.153
33.366	32.834	30.885	33.143	32.566	33.082
33.361	32.364	31.450	33.095	32.684	33.249
33.354	29.438	30.330	32.546	32.505	33.303
33.316	34.969	33.725	34.267	33.900	33.550
33.301	34.515	33.336	33.954	33.372	33.921
33.363	32.148	33.057	33.671	33.683	33.474
33.323	32.927	33.473	32.652	33.434	33.817
33.316	33.199	33.158	32.925	33.166	33.341
33.370	32.103	32.146	33.192	33.359	34.114
33.361	32.782	33.001	33.835	33.880	33.898
33.305	30.496	31.895	33.372	33.488	33.144
33.353	29.738	31.613	32.679	33.618	33.292
33.276	31.913	33.316	31.731	33.491	34.162
33.292	32.808	33.966	32.547	33.898	34.244
33.355	33.706	32.518	33.465	32.486	34.095
33.355	32.425	32.289	32.417	32.450	33.971
33.355	32.707	33.816	33.265	33.893	32.317
33.355	33.036	33.414	33.224	33.394	32.559
33.333	33.080	34.341	32.779	33.712	33.253
33.326	34.949	34.986	34.266	34.223	33.412
33.353	33.112	33.283	32.501	32.555	33.437
33.305	32.803	34.392	31.983	33.564	33.255
33.361	34.911	34.997	34.115	34.216	32.561
33.370	33.745	33.231	33.292	32.529	32.415
33.292	32.989	34.163	33.200	33.770	31.458
33.276	32.922	34.559	33.608	34.147	31.115
33.301	33.696	32.521	33.972	32.474	32.814
33.301	34.554	33.174	33.968	32.510	33.773
33.331	32.488	33.962	32.712	33.859	30.765
33.363	33.482	34.289	32.760	33.673	31.844
33.350	32.284	31.538	32.319	31.392	31.976

	33.365	32.211	31.886	32.281	31.449	33.351
	33.354	32.864	33.010	32.299	32.749	33.407
	33.361	32.237	33.069	32.209	32.850	33.406
	33.316	32.099	31.617	32.160	31.837	33.279
	33.316	31.604	33.028	31.284	32.807	31.438
	33.331	33.895	34.092	33.769	33.726	33.451
	33.332	33.962	34.492	33.820	34.092	33.137
	33.341	32.122	31.760	33.669	33.437	33.126
	33.317	29.950	30.191	32.342	32.456	33.696
	33.301	33.677	32.134	33.975	33.327	33.322
	33.326	33.225	33.011	33.786	33.874	33.559
	33.333	32.734	33.108	33.261	33.720	33.563
	33.366	33.723	31.529	33.386	31.408	33.790
	33.318	32.211	31.618	32.214	31.824	31.467
	33.354	33.738	31.918	33.224	31.472	31.906
	33.316	33.223	32.244	33.756	32.419	32.413
	33.316	32.896	33.171	33.326	33.146	31.932
	33.329	34.912	33.226	34.045	32.528	31.421
	33.323	33.049	33.487	33.217	33.421	29.885
	33.332	32.515	33.315	32.709	33.429	29.559
	33.317	31.673	32.942	31.292	32.707	30.151
	33.341	33.527	34.361	32.737	33.499	30.041
Average	33.3331	32.5362	32.5592	32.9875	33.0023	32.8827
δC^d	151.2605	152.0574	152.0344	151.6061	151.5913	151.7109

a C_{60} at geometry of $CH_2O@C_{60}$ 56 iso. b C_{60} at geometry of $CH_2O@C_{60}$ 56 iso. c $CH_2O@C_{60}$ where C_{60} is fixed at the empty geometry. d Shift corrected to δ scale using a calculated average isotropic ^{13}C chemical shift of Me_4Si carbons of 184.5936.

Supplementary Table 13. Summary of ^{13}C calculated Chemical shift changes on CH_2O incorporation.

	δC / ppm	change from C_{60} / δ ppm
C_{60}	151.2605	0
$CH_2O@C_{60}$ iso 56	152.0574	0.797
$CH_2O@C_{60}$ iso 66	152.0344	0.774
C_{60} at $CH_2O@C_{60}$ 56 iso geometry	151.6061	0.346
C_{60} at $CH_2O@C_{60}$ 66 iso geometry	151.5913	0.331
$CH_2O@C_{60}$ at empty C_{60} geometry	151.7109	0.450

5. NMR studies on $CH_2O@C_{60}$

NMR experiments were performed at the following magnetic fields: (1) at 16.45 T, carried out using a Bruker Ascend 700 NB magnet fitted with a Bruker AVANCE NEO console and Bruker TCI prodigy 5 mm liquids cryoprobe; (2) at 9.4 T using an Oxford AS400 magnet fitted with a Bruker AVANCE NEO console and a Bruker BBO 5mm liquids probe; (3) at 14 T using a Bruker AS 600 WB magnet fitted with a Bruker AVANCE NEO console and a Bruker TBI 5mm liquids probe.

^{1}H and ^{13}C NMR spectra were referenced to the solvent chemical shifts (1,2-dichlorobenzene-d_4), ^{1}H = 6.93 ppm and ^{13}C = 127.19 ppm. The confidence limits of the absolute chemical shift are dominated by the chemical shift of reference solvents, reported to 2 decimal places.

An approx. 23 mM solution of $CH_2O@C_{60}$ in 1,2-dichlorobenzene-d_4 (ODCB-d_4, Sigma-Aldrich), was prepared by dissolving 14 mg of powdered $CH_2O@C_{60}$ (86.7 % filling) in 0.8 mL of the solvent. The solution was then filtered and degassed by bubbling nitrogen gas through the solution for 10 min.

5.1 ^{1}H spin-lattice relaxation of $^{13}CH_2O@C_{60}$

^{1}H T_1 relaxation measurements of the $^{13}CH_2O@C_{60}$ isotopologue, shown in Supplementary Fig. 21, were acquired on the natural abundance $CH_2O@C_{60}$ sample from the $^{13}CH_2O@C_{60}$ ^{1}H satellites. The ^{1}H T_1 of the $^{13}CH_2O@C_{60}$ satellites is only slightly shorter than the ^{1}H T_1 of the $^{12}CH_2O@C_{60}$, 27.9 ± 0.7 & 28.0 ± 0.8 s (Fig. 21) and 30.65 ± 0.04 s (Fig. 4d in main manuscript) respectively. The slightly shorter ^{1}H T_1 value for the $^{13}CH_2O@C_{60}$ isotopologue is attributed to contributions from ^{1}H-^{13}C dipolar relaxation, which are not present for the $^{12}CH_2O@C_{60}$ isotopologue.

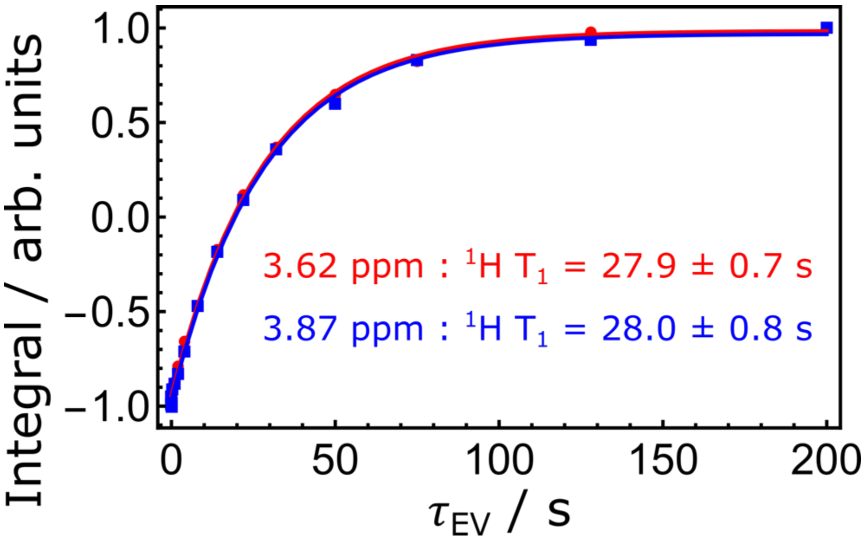

Figure 21 : ^{1}H T_1 relaxation curves (Inversion Recovery) of $^{13}CH_2O$@C_{60} ^{1}H satellites at 3.62 ppm (red) and 3.87 ppm (blue), approx. 23 mM solution in degassed 1,2-dichloro-benzene-*d*$_4$ (ODCB-*d*$_4$) at a field of 16.45 T and 298 K, acquired with 16 transients. ^{1}H T_1 of $^{13}C\underline{H}_2O$@C_{60} satellites: at 3.62 ppm = 27.9 ± 0.7 s and at 3.87 ppm = 28.0 ± 0.8 s.

5.2 ^{13}C spin-lattice relaxation of CH_2O@$\underline{C}_{60}$

The ^{13}C T_1 of CH_2O@$\underline{C}_{60}$ was measured using inversion recovery, shown in Fig 22, to give 17.42 ± 0.06 s. ^{13}C T_1 value is consistent with other measurements of empty and filled C_{60} molecules under equivalent conditions (same solvent and magnetic field).

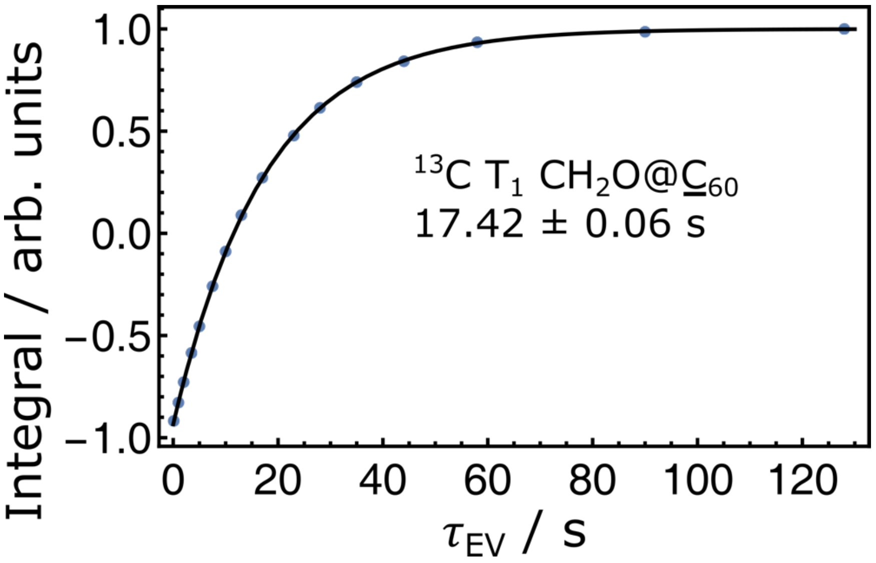

Figure 22: ^{13}C T_1 relaxation curve (Inversion Recovery) of CH_2O@$\underline{C}_{60}$ (86.7 % filling), approx. 23 mM solution in degassed 1,2-dichlorobenzene-*d*$_4$ (ODCB-*d*$_4$) at a field of 16.45 T and 298 K. ^{13}C T_1 of CH_2O@$\underline{C}_{60}$ = 17.42 ± 0.06 s, acquired with 8 transients.

5.3 ^1H spin-lattice relaxation of CH_2O@2 and monomeric CH_2O

Solution state ^1H T_1 relaxation times were measured by inversion recovery in the open-cage intermediate CH_2O@**2** and for monomeric CH_2O in solution. A 33 mM solution of CH_2O@**2** was prepared in ODCB-d_4 (1,2-dichlorobenzene-d_4) and degassed by bubbling with nitrogen gas for 10 minutes. The ^1H T_1 was found to be 2.31 ± 0.03 s (Fig. 23). As free CH_2O rapidly polymerises when dissolved in ODCB, tetrahydrofuran (THF) was used as solvent instead. A 100 mM solution of CH_2O was prepared in 10% THF/90% THF-d_8 and degassed as above. The ^1H T_1 was found to be 30.8 ± 0.8 s (Fig. 24).

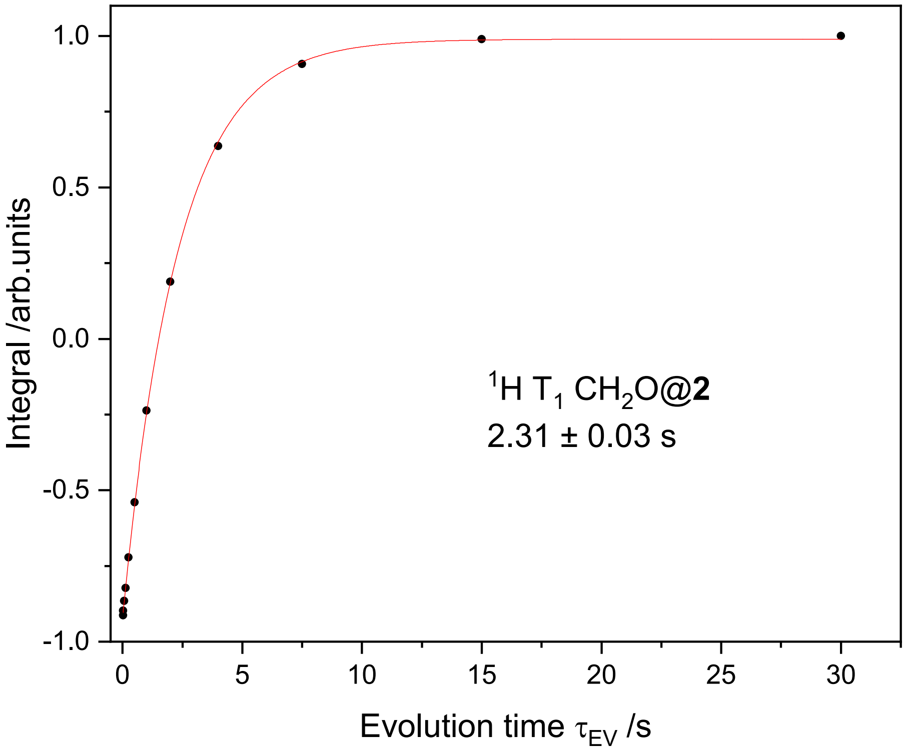

Figure 23: Inversion recovery with experimental data (black circles) and fitted curve (red line) for the spin-lattice relaxation time constant of CH_2O ^1H nuclei in CH_2O@2, 33mM solution in ODCB-d_4. Acquired at 16.45 T and 298 K .

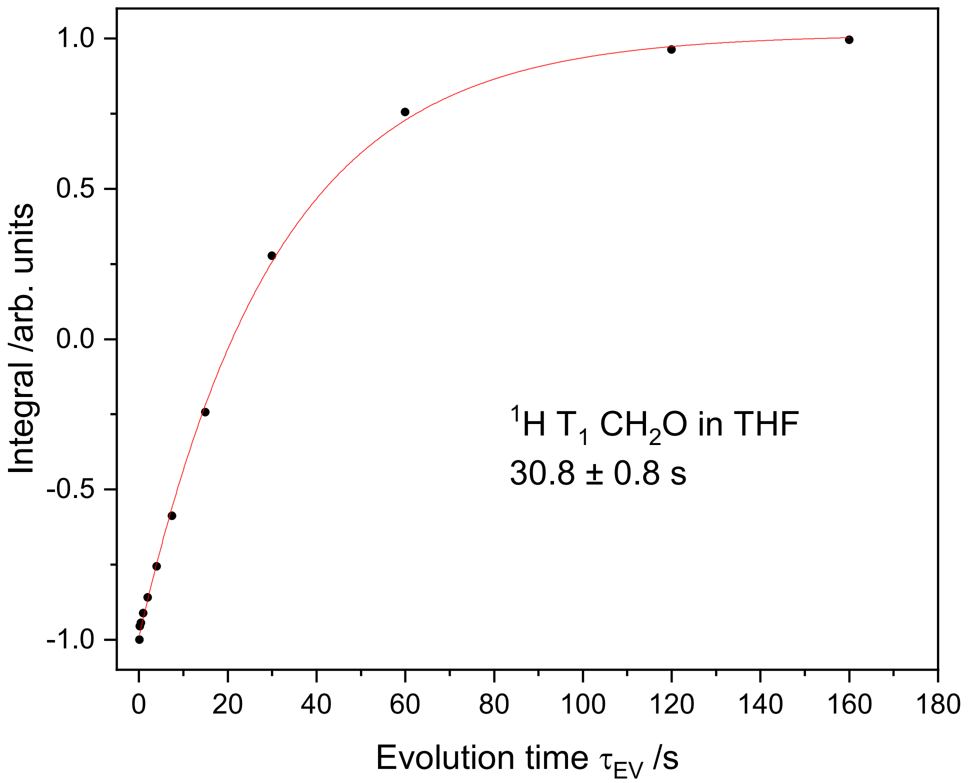

Figure 24: Inversion recovery with experimental data (black circles) and fitted curve (red line) for the ^{1}H spin-lattice relaxation time in free CH_2O, 100 mM solution in 10% THF/90% THF-d_8. Acquired at 16.45 T and 298 K.

6. THz and IR studies on $CH_2O@C_{60}$.

Absorption coefficient was calculated as $\alpha = -d^{-1} \ln(I_s/I_r)$ where I_s and I_r are the light intensities at the detector transmitted by the sample and by a 3 mm diameter reference hole. For the translational modes between 150 and 250cm^{-1}, shown in Fig. 25, the baseline was subtracted. The oscillations seen in the spectrum are due to the interference of IR radiation between the two sample pellet surfaces.

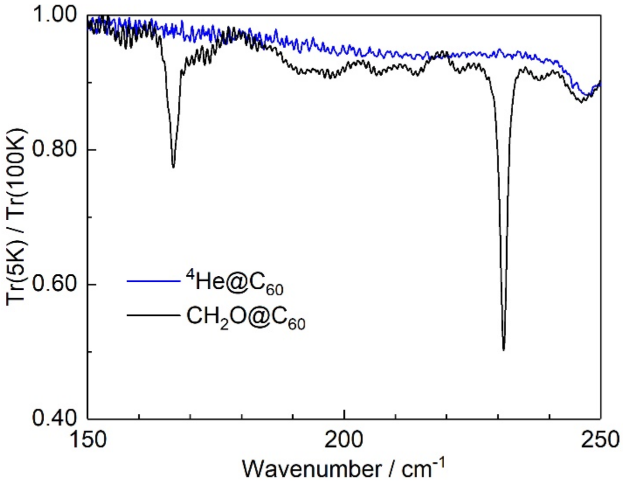

Supplementary Fig. 25. Comparison of transmission spectra of $CH_2O@C_{60}$ (black line) and $^4He@C_{60}$ (blue line). Shown is the ratio of transmission spectrum at 5K to the transmission spectrum at 100K. Absorption lines at 167 and 231cm^{-1} are present in the CH_2O sample but missing in the ^4He sample.

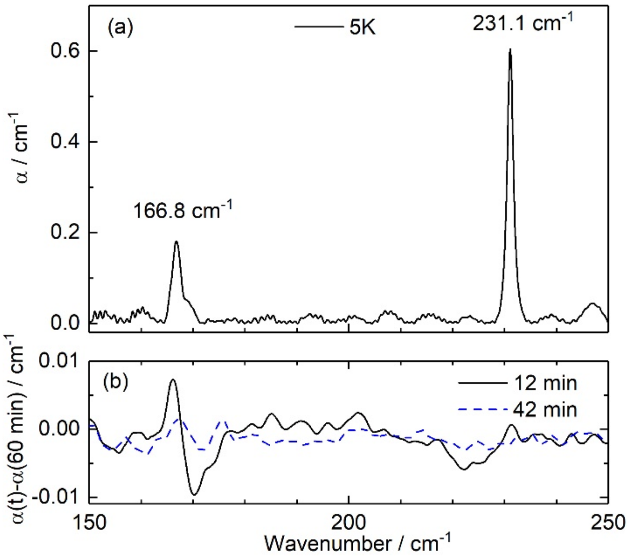

Supplementary Fig. 26 (a) Absorption spectrum of $CH_2O@C_{60}$ at 5K and (b) ortho-para conversion of $CH_2O@C_{60}$ at 5K. Shown is the difference where the spectrum measured at 60 min after the completion of the temperature jump from 20 K to 5 K, subtracted from spectra measured at 12 min and 42 min after the completion of the temperature jump. The positive part in

the difference spectrum is the ortho-CH_2O signal while the negative part is para-CH_2O signal. Absence of ortho-para conversion signature for the 231 cm^{-1} translational mode in the difference spectra could be caused by the overlap of ortho and para species spectral lines for this mode.

7. X-ray structure of $CH_2O@C_{60}$ nickel(II) octaethylporphyrin complex.

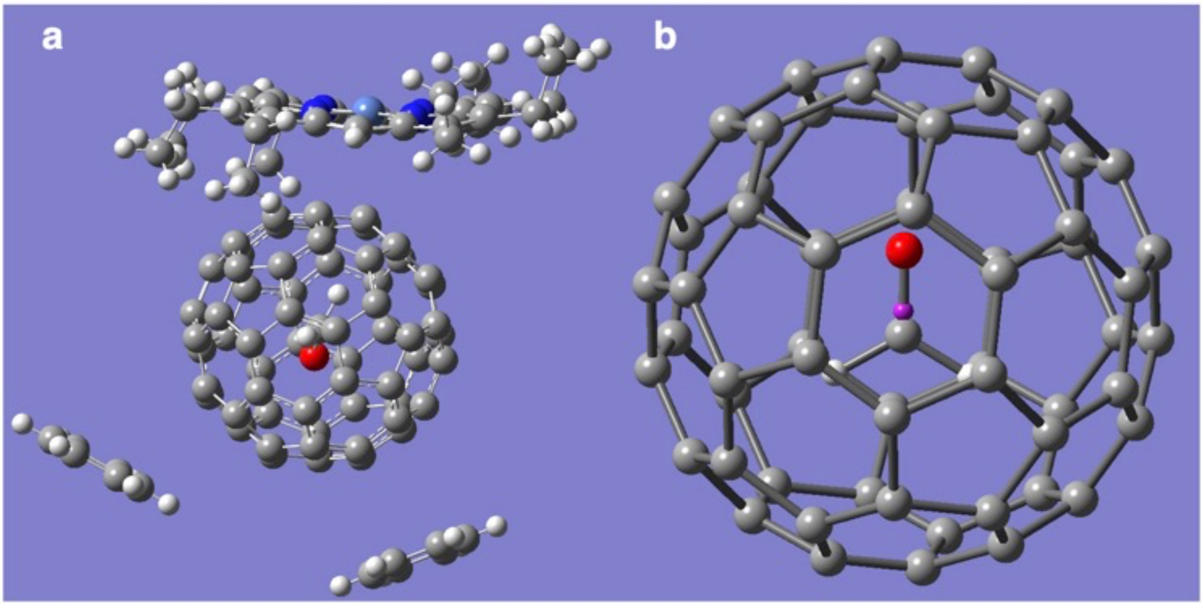

Supplementary Fig. 27. a. Crystal structure of the nickel(II) octaethylporphyrin/benzene solvate of $CH_2O@C_{60}$ with CH_2O position minimised using DFT (B3LYP-D3/6-31(d)) all other atoms being frozen. b. Expansion of cage showing its centroid as purple sphere. Centroid–$\underline{C}H_2$ and centroid-O distances are 0.34 and 0.86Å respectively.

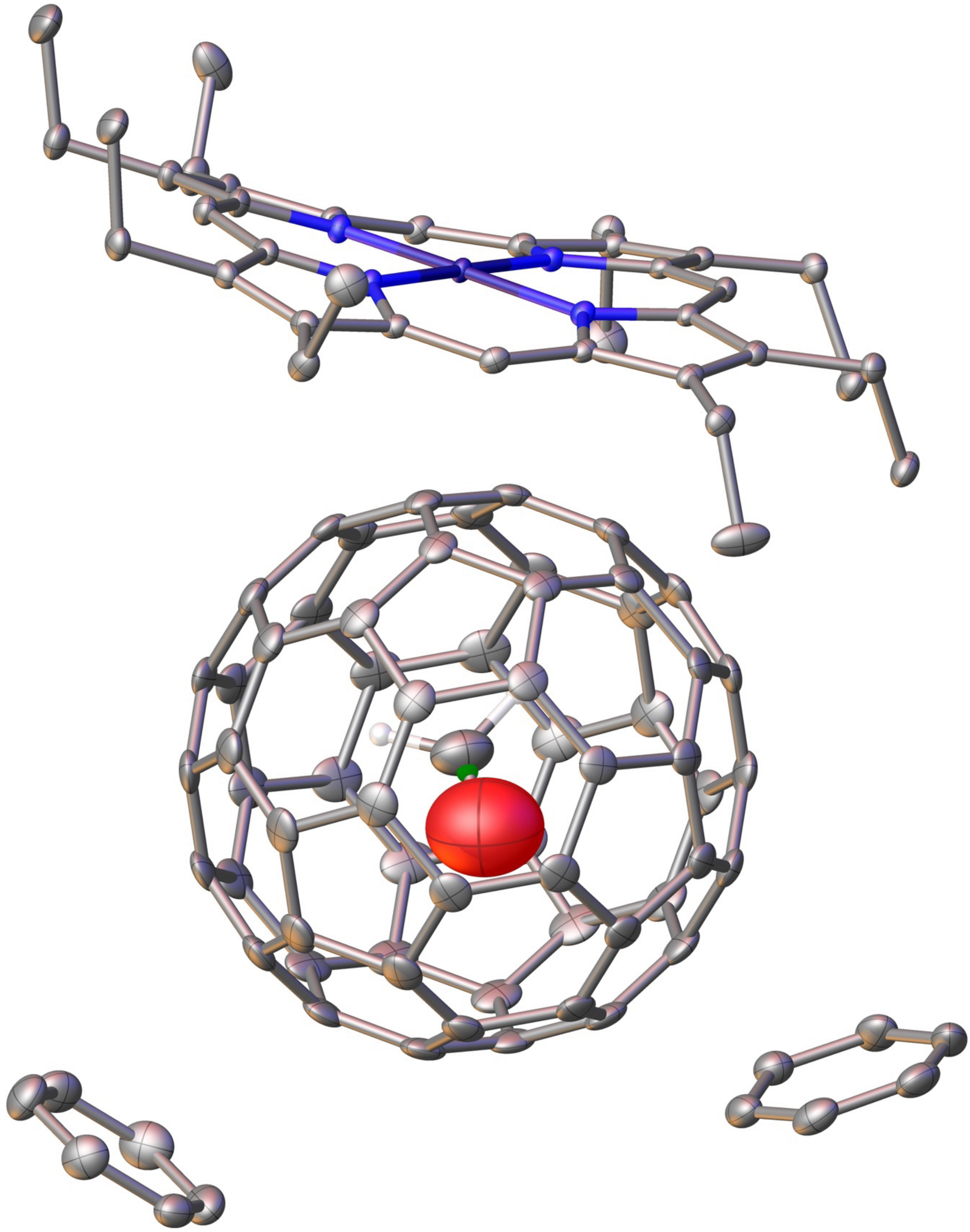

Supplementary Fig. 28. Single crystal x-ray structure of the nickel(II) octaethylporphyrin/benzene solvate of $CH_2O@C_{60}$ at 100 K with CCDC deposition number 2126579 (R1=0.050). Thermal ellipsoids drawn at 50% probability, hydrogens, except on the CH_2O, are omitted for clarity.

8. Photochemistry apparatus.

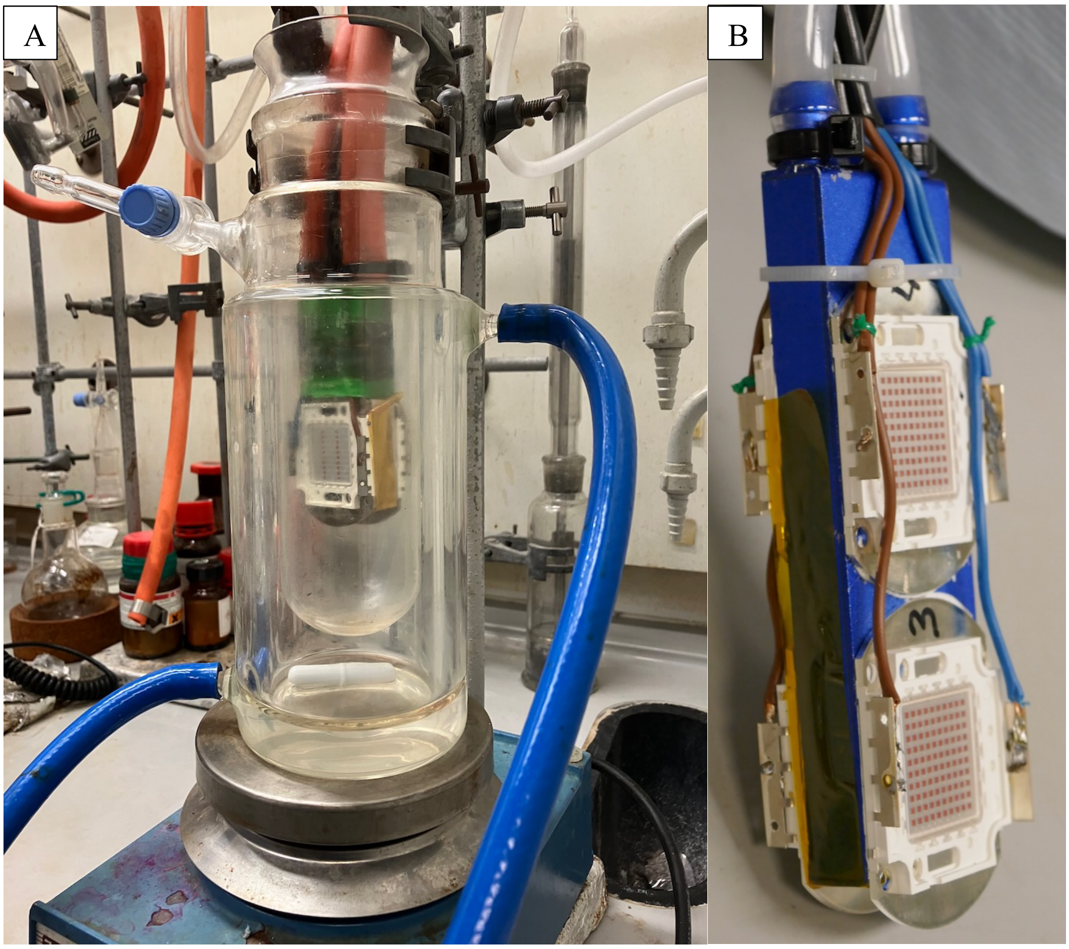

Supplementary Fig. 29. Custom built photochemistry apparatus used for SO extrusion reaction. A Apparatus showing outer jacket for temperature control using a circulating liquid. Middle stirred volume for reaction mixture and inner well containing LED lights (in this picture 2 x 20W). B. The 4 x 100W LED lamp used in the paper. The 100W LEDs were from Chanzon with emission range 590-995 nm (yellow) each driven by a 3A 20-24V constant current driver. The measured power consumption was typically 70W per LED chip. Three LED chips were used in the described reactions. The LEDs are mounted on a water cooled aluminium block.

9. References.

1. Futagoishi, T., Murata, M., Wakamiya, A. & Murata, Y. Encapsulation and Dynamic Behavior of Methanol and Formaldehyde inside Open-Cage C_{60} Derivatives. *Angew. Chem. Int. Ed.* **56,** 2758-2762 (2017).
2. Boys, S. F. & Bernardi, F. The calculation of small molecular interactions by the differences of separate total energies. Some procedures with reduced errors. *Mol. Phys.* **19**, 553-566 (1970).
